# A distinct subset of stem-cell memory is poised for the cytotoxicity program in CD4^+^ T cells in humans

**DOI:** 10.1126/sciadv.ady6423

**Published:** 2026-01-07

**Authors:** Raunak Kar, Shreya Sinha, Zainab Khatun, Anjali Sharma, Veena S. Patil

**Affiliations:** ^1^ Immunogenomics Lab, National Institute of Immunology, New Delhi, Delhi, India.; ^2^ Department of Transfusion Medicine and Pathology, Vardhman Mahavir Medical College and Safdarjung Hospital, New Delhi, Delhi, India.

## Abstract

The CD4^+^ cytotoxic T lymphocytes (CD4-CTLs) with cytotoxic potential are reported to be the components of protective immune response in many diseases. However, the lack of understanding about their lineage, molecular character, and cytolytic potential in comparison to CD8^+^(CD8)-CTLs has restricted their utility. Thus, here, by parallelly analyzing the human peripheral CD4-CTLs and CD8-CTLs, we demonstrate that they are indistinguishable for the cytotoxic program. Furthermore, using an integrative multiomics approach combining the transcriptome, T cell antigen-receptor repertoire, and open chromatin profile of CD4^+^ T cell memory subsets, we found a stem-cell memory subset that is precommitted to the cytotoxicity program. Through an in vitro differentiation model, we developed CD4^+^ T cells with cytolytic potential coexpressing and exhibiting progressive chromatin accessibility for cytotoxicity- and longevity-associated genes, hence generating long-lived CD4-CTL effectors of varying cytotoxic capacity. Together, our study advocates for exploring both CD4-CTLs and CD8-CTLs for vaccine development, vaccine efficacy testing, and immunotherapies and cell-based therapies for precision medicine.

## Teaser

A subset of CD4^+^ memory T cells is indistinguishable from memory CD8^+^ killer T cells, which find their origin in stem-cell memory.

## INTRODUCTION

The naïve T (T_N_) cells activated during the primary infection, in addition to differentiating to effector cells, also form a pool of memory T cells that have the potential to elicit a quicker and stronger immune response against the same pathogen during a secondary infection. The T cell memory pool is heterogeneous and can be further classified on the basis of longevity, proliferative capacity, and effector-ness. Early studies described memory as either long-lived central memory (T_CM_) or short-lived effector memory (T_EM_) and effector memory expressing CD45RA (T_EMRA_) based on the surface expression of cell adhesion molecule, L-selectin (CD62L); isoform of tyrosine phosphatase CD45, CD45RA; costimulatory molecule, CD27; and chemokine receptor type 7 (CCR7) within the CD4^+^ and CD8^+^ T cell subsets in humans (*1*–*3*). Many years later, the antigen-experienced stem-like memory [T stem-cell memory (T_SCM_)] was found within the previously described naïve compartment based on the surface expression of CD95 and interleukin-2 receptor subunit β (IL-2RB) (*4*, *5*). These memory subsets define developmental stages in the memory formation and are better described for the CD8^+^ T cell subset where a more linear development from naïve (T_N_) → T_SCM_ → T_CM_ → T_EM_ → T_EMRA_ has been suggested (*5*). Furthermore, the CD4^+^ T helper (T_H_) memory cells can be classified on the basis of their functional properties into T_H_1, T_H_2, T_H_17, T_H_1/17, T follicular helper (T_FH_), and regulatory T (T_reg_) cells, where each of them have a specialized function (*6*, *7*). T-bet–expressing T_H_1 cells that secrete interferon-γ (IFN-γ) are known for their role in viral infections, and BCL-6 (B cell lymphoma 6)–expressing CD4^+^ T_FH_ cells can secrete interleukin-21 (IL-21) and assist B cells (*6*, *7*). The CD4^+^ helper T cells can also be regulators of immune response via the T_reg_ cells that express forkhead box P3 (FOXP3) and secrete transforming growth factor–β and IL-10 cytokines (*8*). Although the functional CD4^+^ T_H_ memory subsets are relatively well defined, comprehensive studies describing the developmental memory subsets for their lineage, T cell antigen receptor (TCR) clonal expansion, and epigenetic program are far fewer (*9*–*11*). Each T_H_ memory subset can exist in both long-lived and effector memory subsets, suggesting their different developmental stages (*7*).

Although, classically, the CD4^+^ T cells are known for their helper roles and the CD8^+^ T cells for their cytotoxic roles in immune responses, the major histocompatibility complex II–restricted CD4^+^ T cells have also been shown to be killers in a wide array of infectious diseases, cancers, and autoimmune disorders for many decades now [reviewed in (*12*)]. The circulating CD4^+^ cytotoxic T lymphocytes (CD4-CTLs) have been better studied in viral infections in both humans and animal models, which include human cytomegalovirus, Epstein-Barr virus, human immunodeficiency virus (HIV), dengue virus, influenza virus, severe acute respiratory syndrome coronavirus 2 (SARS-CoV2), and many more [reviewed in (*12*)]. The magnitude of the CD4-CTL response has been associated with better clinical outcomes in both acute and chronic viral infections and antitumor immune responses, although a few reports suggest their protumoral roles and potential pathogenic role in SARS-CoV2 (*13*–*15*). Successful vaccinations against yellow fever virus, influenza virus, smallpox, vaccinia virus, poliovirus, and HIV have also been shown to elicit CD4-CTL responses (*12*, *13*, *16*, *17*). Thus, eliciting a strong CD4-CTL response has been considered an important goal of vaccination against several viral infections.

The CD4-T_EMRA_ memory compartment has been shown to be enriched for CD4-CTLs (*9*, *10*, *18*). However, the molecular and epigenetic landscapes that drive the differentiation, maintenance, and function of human CD4-CTLs from T_N_ cells are still elusive. Furthermore, although it has been shown that CD4-T_EMRA_ expresses cytotoxicity-associated genes and shows enrichment for the overall cytotoxic program, it has not been well studied in conjunction with the bona fide cytotoxic T cells, the CD8-CTLs (CD8-T_EMRA_), to assess their cytolytic potential (*9*). Hence, in this study, through global transcriptomics- and cytotoxicity-related protein expression analysis from the same donors, we show that CD4-CTLs and CD8-CTLs are indistinguishable for the cytotoxicity program, and both show increased TCR clonal expansion, the mark of the effector status of these cells. Next, using both bulk and single-cell transcriptomic analysis paired with TCR analysis and open chromatin analysis, we identified T_SCM-CTL_ cells, stem-like memory cells poised for the CD4-CTL lineage within the CD4-T_SCM_ subset. Furthermore, through an in vitro differentiation model, we systematically developed CD4^+^ T cells with a cytolytic ability from CD4-T_N_ cells, established a differentiation pathway, and identified transcriptomic as well epigenomic regulators of the CTL program development that overlaps with ex vivo CD4-T_EMRA_ effector cells, thus establishing the CD4-CTL lineage from naïve cells via the T_SCM-CTL_ cells.

## RESULTS

### The cytolytic program of CD4-CTLs and CD8-CTLs is indistinguishable

**Fig. 1.**
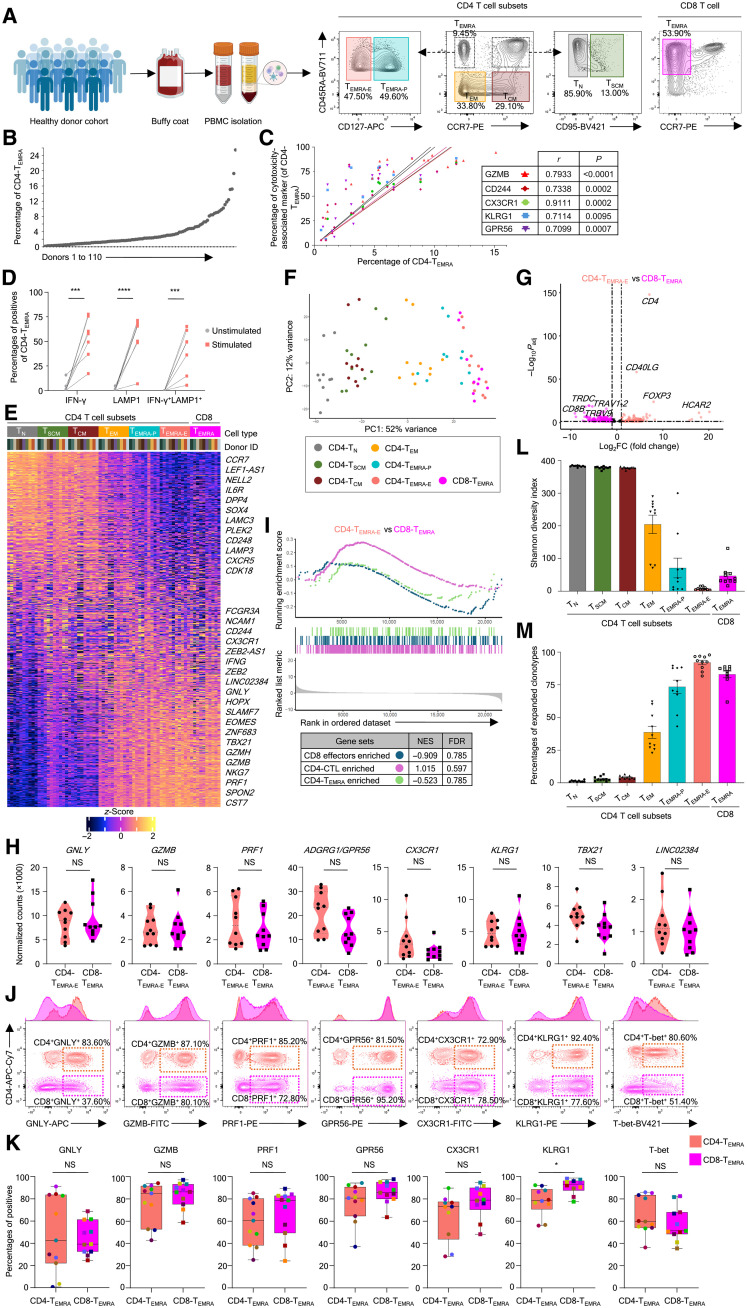
The CD4-T_EMRA_ subset resembles the CD8-T_EMRA_ subset. (**A**) Schematic representation of study design. The percentages are of parent population. Created in BioRender. V. S. Patil (2025); https://biorender.com/w0129ho. (**B**) The scatterplot shows the proportions of CD4-T_EMRA_ of CD4^+^ T cells. (**C**) The scatterplot of Pearson correlation between the proportion of CD4-T_EMRA_ and the proportion of CD4-T_EMRA_ positive for the indicated cytotoxicity-related molecules. Each colored line represents the linear regression of correlation with a 95% confidence interval (*n* = 16 to 20). The correlation *r* value and *P* value from Student’s paired two-tailed *t* test for each molecule are tabulated. (**D**) The matched scatterplot shows the percentage of IFN-γ^+^, LAMP1^+^, and IFN-γ^+^LAMP1^+^ cells in the CD4-T_EMRA_ compartment (*n* = 7). Error bars represent the mean ± SEM. ***P < 0.005, and ****P < 0.001 from Student’s paired two-tailed t test. (**E** and **F**) The heatmap of bulk transcriptomic analysis shows the row-wise *z*-score of normalized counts (E) and PCA plot (F) of 2000 most variable genes across the indicated cell types from 10 donors. (**G**) Volcano plot for differentially expressed transcripts (based on DESeq2) between CD4-T_EMRA-E_ (peach) and CD8-T_EMRA_ (magenta). (**H**) Violin plots show the normalized counts for the indicated transcripts. NS, nonsignificant based on DESeq2 between CD4-T_EMRA-E_ and CD8-T_EMRA_. (**I**) Combined GSEA plot for indicated gene sets comparing CD4-T_EMRA-E_ and CD8-T_EMRA_. Individual normalized enrichment scores (NESs) and false discovery rates (FDRs) are tabulated. (**J**) Representative contour plots show the expression of the indicated proteins within the CD4-T_EMRA_ (peach) and CD8-T_EMRA_ (pink) compartments in a flow cytometry analysis. Adjacent histograms show the geometric mean fluorescence intensity. Percentages are of parent population. (**K**) Box plots with a median and interquartile range show the percentage of positives for the indicated proteins within the mentioned subsets in a flow cytometry analysis (*n* = 9 to 11). *P < 0.05; NS, *P* > 0.05 from Student’s paired two-tailed *t* test. (**L** and **M**) Bar graphs show the mean Shannon-Weiner diversity index (L) and proportions of expanded clonotypes (≥3) (M) for TCRβ clonotypes (*n* = 10).

The CD4-CTLs have been shown to be enriched in the CD4-T_EMRA_ (effector memory expressing CD45RA; CD4^+^CD45RA^+^CCR7^−^) memory compartment, which was further found to be heterogeneous with mixtures of effectors and precursors of CD4-CTLs, which could be distinguished on the basis of IL-7R (CD127) expression (*9*, *10*). The proportion of CD4-T_EMRA_ varies greatly across individuals and is positively correlated with a better clinical outcome in viral infections and vaccination (*9*, *10*, *17*–*19*). Consistent with a previous report, we observed a huge variability in the CD4-T_EMRA_ proportion, ranging from 0.11% to more than 25% of the total CD4^+^ T cells in the peripheral blood across 110 subjects (Fig. 1, A and B) (*9*). We also noted a significant positive correlation between the frequency of CD4-T_EMRA_ and the proportion of CD4-T_EMRA_ expressing cytotoxicity-associated molecules such as granzyme B (GZMB), CD244 (2B4), C-X3-C motif chemokine receptor 1 (CX3CR1), killer cell lectin-like receptor G1 (KLRG1), and G protein (heterotrimeric guanine nucleotide–binding protein)–coupled receptor 56 (GPR56; (ADGRG1) (Fig. 1C and table S1) (*9*, *10*, *13*, *18*). Similarly, upon stimulation, the CD4-T_EMRA_ cells produced IFN-γ, a cytokine made by cytotoxic effector T cells, and expressed the degranulation marker lysosomal-associated membrane protein 1 (LAMP1) on the cell surface, further highlighting the potential cytolytic ability of these cells (Fig. 1D and fig. S1A) (*9*, *10*, *18*, *20*). Hence, to identify and understand the overall cytotoxic program of CD4-CTLs in relation to the other CD4^+^ T memory and T_N_ cell subsets, we performed RNA sequencing (RNA-seq) of ex vivo–isolated CD4^+^ T cell memory compartments, CD4-T_N_ (T_N_ cells; CD4^+^CD45RA^+^CCR7^+^CD95^−^), CD4-T_SCM_ (stem-cell memory; CD4^+^CD45RA^+^CCR7^+^CD95^+^), CD4-T_CM_ (central memory; CD4^+^CD45RA^−^CCR7^+^), CD4-T_EM_ (effector memory; CD4^+^CD45RA^−^CCR7^−^), CD4-CD127^hi^ T_EMRA_ (precursor CD4-CTL or CD4-T_EMRA-P_), and CD127^lo^ CD4-T_EMRA_ (effector CD4-CTL or CD4-T_EMRA-E_), from 10 healthy human subjects with a higher CD4-T_EMRA_ proportion (Fig. 1, A, B, E, and F, and fig. S1, B to D) (*1*, *4*, *5*, *9*). The transcriptomic data analysis using the 2000 most variable transcripts showed that the T_SCM_ and T_CM_ subsets resembled that of T_N_ cells and showed enrichment for signatures of longevity, while the T_EM_ and precursor- and effector-T_EMRA_ subsets clustered together in a principal components analysis (PCA) and showed enrichment for effector molecules (*GZMB*, *PRF1*, *CD244*, *CX3CR1*, *FCGR3A*, *IFNG*, etc.) (fig. S1, C and D). We then compared the transcriptomes of the CD4-CTLs (CD4-T_EMRA-P_ and CD4-T_EMRA-E_) with CD8-CTLs (CD8-T_EMRA_: CD8^+^CD45RA^+^CCR7^−^) from the same donors. We observed that they clustered together in a PCA and expressed cytotoxicity-associated transcripts at comparable levels, indicating shared transcriptomic signatures between CD4-CTLs and CD8-CTLs (Fig. 1, A, E, and F). Even the pairwise comparison of CD4-T_EMRA_ and CD8-T_EMRA_ subsets did not identify any notable differences in the expression of transcripts associated with the cytotoxicity program (Fig. 1, G and H; fig. S1E; and data file S1). The expression of the cytotoxicity-associated molecules such as *GZMB*, *GNLY*, *PRF1*, *ADGRG1* (*GPR56*), *KLRG1*, *CX3CR1*, and transcription factors (TFs) and the long noncoding RNA associated with CTLs, *TBX21* (encoding T-bet) and *linc02384*, was comparable between CD4-CTLs and CD8-CTLs (Fig. 1H and data file S1) (*9*). The gene set enrichment analysis (GSEA) using the known gene sets enriched in CD8 effectors, CD4-CTL effectors, and CD4-T_EMRA_ also showed no significant differences between CD4-CTLs and CD8-CTLs (Fig. 1I and data file S2) (*9*, *21*, *22*). Next, to assess whether the differences could be captured at the protein level, we performed flow cytometry analysis to examine the expression of various proteins and TFs associated with cytotoxicity [granulysin (GNLY), GZMB, perforin 1 (PRF1), GPR56 (ADGRG1), CX3CR1, KLRG1, and T-bet] between CD4-CTLs and CD8-CTLs (Fig. 1J). Even at the protein level, most of these CTL-associated molecules showed no significant differences between CD4-T_EMRA_ and CD8-T_EMRA_ (Fig. 1, J and K). Overall, these results show that there are no notable differences between CD4-CTL and CD8-CTL memory subsets, and both share similar gene expression profiles.

The T cells are known for the diversity in the TCR clonotype with every cell having a unique clonotype, unless they have resulted from clonal expansion in response to an infection or an immunological event; hence, effector cells are known to show a restricted TCR repertoire (*23*). Thus, to understand the TCR clonal diversity and clonal expansion across the memory subsets, we performed TCR sequencing (TCR-seq) analysis of both TCRα and TCRβ chains across CD4^+^ naïve and memory subsets and CD8-T_EMRA_ (*24*, *25*). TCR repertoire analysis showed that similar to T_N_ cells, the long-term memory subsets T_SCM_ and T_CM_ have a highly diverse TCRα and TCRβ repertoire, while the effector memory subsets have limited diversity (Fig. 1L, fig. S1F, and data file S3). On the other hand, like the CD8-T_EMRA_ subset, the CD4-T_EMRA_ subsets are hugely clonally expanded (Fig. 1M, fig. S1G, and data file S3) (*9*). We also noted preferential usage of a few V and J genes over others that could not be correlated with their genomic locus in both TCRα and TCRβ clonotypes across the memory subsets, thus highlighting the random selection of V and J genes during recombination at the TCR loci (fig. S1H and data file S3). The single-cell transcriptomic and TCR repertoire analysis of rare human cytomegalovirus–specific memory T cells has also revealed similar observations (*26*). Together, these data show that the CD4-CTL and CD8-CTL memory compartments are very similar for their cytotoxicity program and are the result of clonal expansion, indicating that the program used for cytolysis by these cells is the same and the difference potentially comes from how they recognize the pathogen.

### CD4^+^ T_SCM_ shows dual signatures

**Fig. 2.**
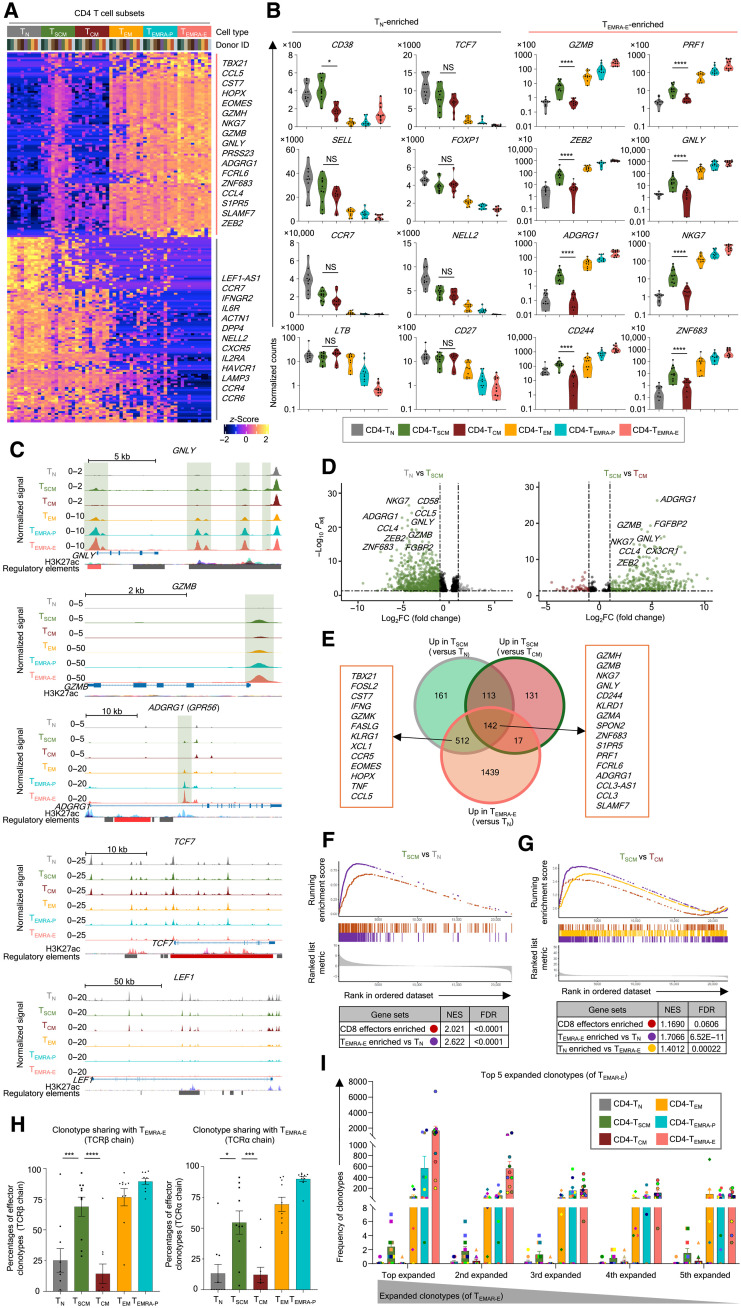
CD4^+^ T_SCM_ shows dual signatures. (**A**) The heatmap of bulk transcriptomic analysis shows the row-wise *z*-score normalized expression of top 100 (each) significantly up-regulated transcripts based on DESeq2, between T_N_ and T_EMRA-E_, visualized across the indicated CD4^+^ T cell compartments from 10 donors. (**B**) Violin plots of normalized counts for indicated T_N_-enriched (versus T_EMRA-E_) and T_EMRA-E_-enriched (versus T_N_) transcripts across the indicated CD4^+^ T cell compartments (*n* = 10 donors). Benjamini-Hochberg *P_adj_ < 0.05, ****P_adj_ < 0.001, and NS *P_adj_* > 0.05 from DESeq2 analysis. (**C**) Coverage plots of indicated genes show the normalized signal intensity of peaks across the gene body, aggregated for 10 donors (*n* = 10) generated using bulk ATAC-seq. GeneHancer regulatory elements (Double Elite) show putative regulatory elements (red, promoter; gray, enhancer). Layered H3K27ac marks for the corresponding genomic loci from ENCODE are shown as a peak (histogram) plot. Peaks of interest are highlighted in green. (**D**) Volcano plots for pairwise-differentially expressed transcripts based on DESeq2 between T_N_ versus T_SCM_ (left) and T_SCM_ versus T_CM_ (right), colored on the basis of the subset where they are up-regulated. (**E**) The Venn diagram shows the transcripts enriched in T_SCM_, T_CM_, and T_EMRA-E_ subsets from the indicated pairwise comparisons using DESeq2. Examples of overlapping genes are shown in boxes. (**F** and **G**) Combined GSEA plots show the enrichment of the indicated gene sets in T_SCM_ compared to T_N_ (F) and T_CM_ (G). Individual NES and FDR are tabulated. (**H**) Bar graphs show the percentage of the expanded clonotypes [≥3, TCRβ (left) and TCRα (right)] from T_EMRA-E_ shared by the indicated CD4 T cell subsets. Error bars represent the mean ± SEM. *P < 0.05, ***P < 0.005, and ****P < 0.001 from Student’s paired two-tailed *t* test. (**I**) The bar graph shows the frequency of the top five expanded clonotypes from the T_EMRA-E_ subset across the CD4^+^ memory T cell subsets. *n* = 10 [(H) and (I)].

Considering the nonclassical cytotoxic nature of CD4^+^ T cells in the CD4-T_EMRA_ compartment, we next wanted to delineate the developmental trajectory of CD4-CTLs by identifying a potential long-lived memory subset that is precommitted to the CTL lineage in the CD4^+^ memory T cell subsets. To this end, we first compared the expression of transcripts that distinguish CD4-T_N_ and CD4-T_EMRA-E_ across all the developmental CD4^+^ T cell memory subsets (Fig. 2A and data file S1). Consistent with our previous observation, largely the long-term memory subsets (T_SCM_ and T_CM_) resembled CD4-T_N_, while the effector memory subsets (T_EM_ and T_EMRA-P_) resembled CD4-T_EMRA-E_ and clustered as two groups in the PCA (Fig. 2A and figs. S1, C and D, and S2A). The transcripts such as *TCF7*, *SELL*, *FOXP1*, *CCR7*, *NELL2*, *LTB*, and *CD27* that were up-regulated in CD4-T_N_ compared to T_EMRA-E_ were also expressed at an elevated level by T_SCM_ and T_CM_ when compared to effector memory, although the expressions were further reduced in them compared to CD4-T_N_ (Fig. 2, A and B, and data file S1). The T_SCM_ subset could be further distinguished from CD4-T_N_ on the basis of the expression of *FAS* (*CD95*), *CXCR3*, *IL2RB*, and *CD58*, which represents the status of antigenic experience (fig. S2B) (*4*, *5*). The T_SCM_ subset was also marked by the highest expression of the stem-cell marker *CD38* (Fig. 2B) (*27*). Similarly, the transcripts up-regulated in effectors (*GZMB*, *PRF1*, and *NKG7*) were also expressed by T_EM_ and T_EMRA-P_ subsets, albeit at a reduced level compared to T_EMRA-E_ (Fig. 2, A and B) (*9*).

Next, to understand the open chromatin status of these memory cells, which can assist in understanding the regulation of gene expression, we performed ATAC-seq (assay for transposase-accessible chromatin using sequencing) on all six CD4^+^ T cell subsets in the same 10 donors (Fig. 2C and fig. S2, C to E). The ATAC-seq data analysis of CD4^+^ T cell subsets revealed that the variable peaks were distributed across various genomic regions with the highest number of peaks falling in intronic regions (~59%) followed by distal intergenic regions (~26%) (fig. S2, C and D). These variable peaks largely segregated the memory compartments into long-term and effector memory subsets (fig. S2E). The peaks associated with genes (e.g., *TCF1* and *LEF1*) expressed in long-term memory had the highest accessibility in T_N_ (naïve cells) where the intensity decreased from T_N_ to T_SCM_ to T_CM_ (Fig. 2C and fig. S2D). On the other hand, the effector-associated peaks (e.g., *GZMB*, *GNLY*, and *GPR56*) had the highest accessibility in T_EMRA-E_, which gradually decreased in T_EMRA-P_ followed by T_EM_ (Fig. 2C and fig. S2D). The overall open-chromatin landscape of the CD4^+^ T cell memory subsets suggests a gradual change in memory phenotype where the long-term memory–associated peaks slowly close as we go from T_N_ cells to developmentally more mature cells such as T_EMRA-E_, while the effector-associated peaks gain accessibility.

Among the long-term memory subsets, we noted a significantly higher expression of the cytotoxicity-associated transcripts (*GZMB*, *NKG7*, *PRF1*, *GNLY*, *ADGRG1*, *CD244*, *ZEB2*, and *ZNF683*) in T_SCM_ compared to both T_N_ and T_CM_, while the expression of the naïve-enriched genes such as *CD38*, *TCF7*, *SELL*, *FOXP1*, *CCR7*, *NELL2*, *LTB*, and *CD27* was either higher or unchanged in T_SCM_ compared to T_CM_ (Fig. 2, A and B, and data file S1). Even in a pairwise comparison, the T_SCM_ compartment showed enrichment for effector-associated genes compared to both T_N_ and T_CM_ (Fig. 2, D and E, and data file S1). Furthermore, the GSEA using the CD8 effector–enriched gene set (*22*) as well as T_N_ (versus T_EMRA_)– and T_EMRA_ (versus T_N_)–enriched gene sets (this study) revealed that T_SCM_ is enriched for both long-term memory– and naïve-associated gene signatures compared to T_CM_ and effector-specific gene signatures compared to both T_CM_ and T_N_ (Fig. 2, F and G, and data file S2). Even in the open-chromatin analysis, we observed that the peaks associated with effector-specific molecules such as *GZMB*, *GNLY*, and *ADGRG1* showed a greater openness in both regulatory regions and gene body in the T_SCM_ subset compared to T_N_ or T_CM_ (Fig. 2C, highlighted region). Together, these results revealed that the T_SCM_ compartment shows dual signatures, where they are both stem-like and effector-like when compared to the other long-term memory subset, T_CM_, hence phenocopying the signatures of naïve cells as well as effector cells. This intriguing observation led us to examine whether T_SCM_ cells are the potential progenitors that develop into the CTL lineage in the CD4^+^ T cell compartment.

T cells have natural barcodes in the form of TCRs, which act as their unique identifiers, enabling one to trace them even when these T cells acquire different phenotypes upon an immunological event. To establish the potential lineage connection between CD4-CTLs and other CD4^+^ memory T cell subsets, we examined the TCR clonotype sharing between T_EMRA-E_ and other naïve and long-term memory subsets using the TCR-seq data. We observed that a significantly higher proportion of the expanded clonotypes of effectors (CD4-CTLs; T_EMRA-E_) was shared by the T_SCM_ compartment as compared to the other long-term memory compartment, T_CM_, and the T_N_ for both TCRβ and TCRα chains (Fig. 2H and data file S3). Even the top five most expanded clonotypes of effectors were found in the T_SCM_ subset, although these clonotypes themselves were not greatly expanded in T_SCM_ (Fig. 2I and data file S3). More than 80 and 70% of the donors shared the top expanded and second most expanded clonotypes, respectively, between T_SCM_ and T_EMRA-E_ (fig. S2F and data file S3). Together, these results suggest a potential developmental connection between T_SCM_ and T_EMRA_ within the CD4^+^ T cell compartment.

### Identification of the T_SCM-CTL_ subset within T_SCM_

**Fig. 3.**
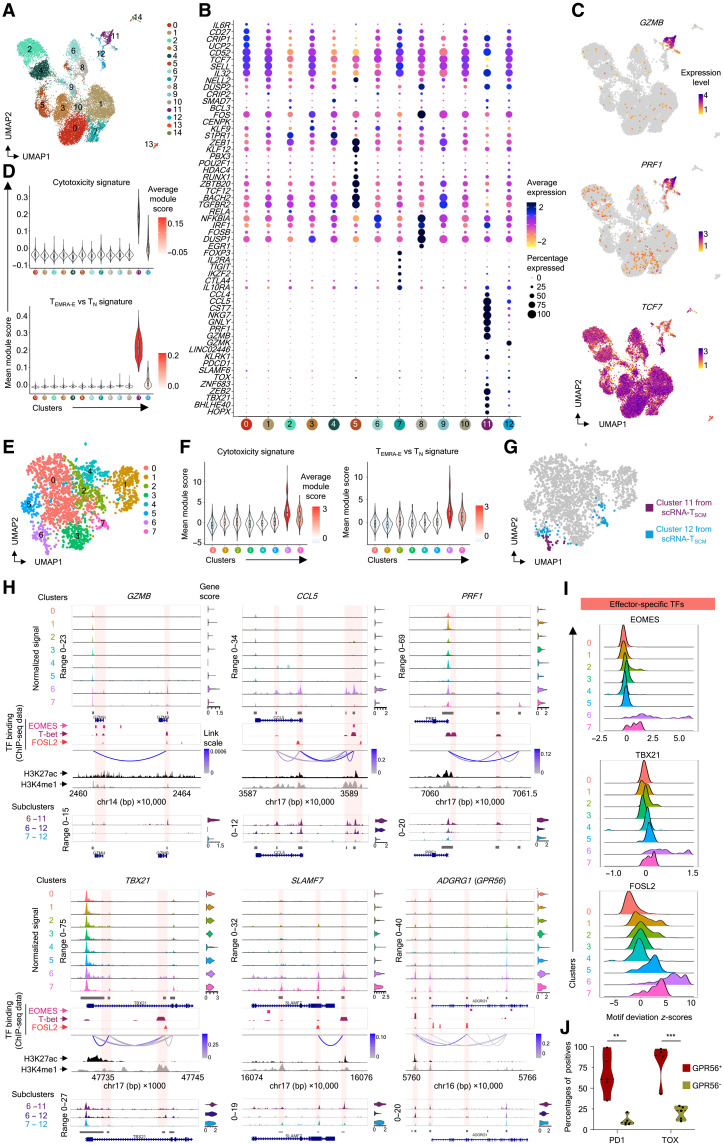
scRNA-seq and scATAC-seq analyses of T_SCM_ cells. (**A**) 2D UMAP projection of the scRNA-seq analysis of 17,364 T_SCM_ cells. (**B**) The dot plot shows the mean expression (color) and percentage of expressing cell (size) of indicated differentially expressed transcripts across clusters. (**C**) 2D UMAP visualization of normalized expressions of indicated transcripts. (**D**) Violin plot for the mean signature scores (*y* axis) for indicated gene modules per cluster (*x* axis) painted by the average score. (**E**) 2D UMAP embedding of scATAC-seq data from 2240 T_SCM_ cells. (**F**) Violin plot for the mean accessibility scores (*y* axis) of the peaks linked to the signature genes for indicated gene modules per cluster (*x* axis) painted by the average score. (**G**) 2D UMAP embedding painted by the predicted identity of clusters 11 and 12 from the scRNA-seq analysis of T_SCM_ projected onto scATAC-seq data. (**H**) Chromatin accessibility of indicated gene-containing genomic loci across indicated clusters/subclusters, visualized using CoveragePlot (top) with the group gene score as violin plots (right) and significant coaccessible peaks connected via links (calculated using cicero) colored on the basis of scores. Publicly available ChIP-seq data for T-bet, EOMES, and FOSL2 for the corresponding genomic loci shown as colored peak plots. ENCODE-sourced histone modification marks of H3K27ac (black) and H3K4me1 (gray) are shown. Peaks or regions of interest are highlighted. (**I**) Ridge plot of chromVAR deviation (*z*-)scores for indicated effector-associated TFs across clusters. (**J**) Violin plots with the median and interquartile range show the percentage of TOX^+^ and PD1^+^ populations within the GPR56^+^ and GPR56^−^ populations in the T_SCM_ subset in a flow cytometry analysis. *n* = 5 donors. **P < 0.01 and ***P < 0.005 from Student’s paired two-tailed *t* test. For (B) and (D), clusters with <1% of the total cells are not shown (cluster 13 = 167 and cluster 14 = 86 cells).

We next wanted to address any potential heterogeneity in the T_SCM_ subset and identify subpopulation(s) of T_SCM_ precommitted to the CD4-CTL phenotype. To interrogate the heterogeneity at both transcriptomic and epigenetic levels, we performed single-cell RNA-seq (scRNA-seq) and single-cell ATAC-seq (scATAC-seq) assays on the T_SCM_ subset isolated from peripheral blood mononuclear cells (PBMCs) of six to eight donors. The scRNA-seq analysis revealed 15 clusters, and as expected, considering the stem-like nature of this subset, most of the clusters expressed long-term memory/naïve–associated transcripts such as *CD27*, *NELL2*, and *TCF7* (encoding TCF1) (Fig. 3, A to C, and data file S4). However, in addition, these cells expressed gene signatures of other functional subsets in a cluster-specific manner (Fig. 3, A and B, and data file S4). For example, cluster 5 showed enrichment for transcripts of TFs known to be expressed by stem-cell/T_N_ cells such as *ZEB1*, *BACH2*, *POU2F1*, *KLF12*, etc., suggesting that these cells are possibly the most undifferentiated cells or show higher stemness features (Fig. 3B and data file S4) (*28*–*31*). Similarly, cluster 7 showed T_reg_-specific transcripts, *FOXP3*, *IL2RA*, *TIGIT*, *IKZF2*, *CTLA-4*, and *IL10RA*, indicating potential stem-cell memory precursors of T_reg_ cells (Fig. 3B and data file S4). Clusters 11 and 12 were enriched for effector-specific transcripts associated with cytotoxicity such as *GZMB*, *GNLY*, and *PRF1* and TFs *ZEB2*, *HOPX*, *ZNF683*, *TBX21*, and *BHLHE40* and showed enrichment for cytotoxicity- and effector-enriched gene sets, hence representing the CTLs in the T_SCM_ subset (hereafter referred to as T_SCM-CTL_ cells) (Fig. 3, B to D; fig. S3A; and data files S2 and S4) (*9*, *14*, *32*–*34*). The presence of T_SCM-CTL_ cells in T_SCM_ was also further validated at the protein level for CTL-associated molecules such as GZMB, PRF1, GNLY, CX3CR1, CD244, and GPR56 (ADGRG1) using flow cytometry analysis (fig. S3B). Consistently, the T_SCM-CTL_ cells constituted a very small fraction of T_SCM_, even in flow cytometry analysis, and were spread across the T_SCM_ subset without showing any bias for either low or high expression of CD45RA and CCR7 (fig. S3B). Although both clusters 11 and 12 showed enrichment for cytotoxicity-associated transcripts compared to other clusters, overall, the expressions were more pronounced in cluster 11 compared to cluster 12, hinting toward probably more than one stage in the CTL development within the T_SCM_ subset (Fig. 3, B to D, and data file S4). Hence, next to understand whether a subset of cells in T_SCM_ is in a poised state for the CTL lineage and to identify the gene-expression regulation patterns of these cells, we performed scATAC-seq and identified eight distinct clusters on the basis of chromatin accessibility (Fig. 3E). Two clusters, cluster 6 and 7, showed the overall higher mean accessibility score for peaks linked to the cytotoxicity-associated, CD4-T_EMRA_–associated, and effector (versus naïve)–enriched gene sets, with cluster 6 showing relatively higher accessibility compared to cluster 7 (Fig. 3F, fig. S3C, and data file S2) (*9*, *35*). On the basis of the prediction scores obtained from an integrative analysis of scRNA-seq and scATAC-seq data, we predicted the cells in clusters 6 and 7 to be the T_SCM-CTL_ cells identified in scRNA-seq (Fig. 3G). We then analyzed the chromatin accessibility of the genomic loci that contained CTL-associated genes across the clusters using genomic tracks and calculated group gene scores that are indicative of gene activity (Fig. 3H, violin plots). Both clusters 6 and 7 showed the overall higher gene activity for *GZMB*, *CCL5*, *PRF1*, *TBX21*, *SLAMF7*, and *ADGRG1* (Fig. 3H, violin plots). Furthermore, we found several peaks in either the gene body or regulatory regions of genes such as *GZMB*, *GPR56* (*ADGRG1*), *CCL5*, *SLAMF7*, and *TBX21* that were differentially accessible, showing relatively higher accessibility in cluster 6 than cluster 7, and several of these peaks overlapped with histone marks associated with gene activation such as H3K4me1 and H3K27ac (highlighted area in Fig. 3H) (*36*–*38*). However, the chromatin accessibility of long-term memory–associated genes such as *CCR7*, *CD27*, *SELL*, *TCF7*, *LEF1*, and *CD28* did not show any noticeable variation across these clusters (fig. S3D). We found several peaks showing positive correlation, implicating coaccessibility, thus suggesting a probable association of these open peaks with gene expression in a co-regulated manner (Fig. 3H) (*39*). For example, peaks upstream of *CCL5*, *SLAMF7*, *ADGRG1*, and *PRF1* in cluster 6 showed a higher correlation with multiple peak/s in the gene body, indicating a possible interaction of these genomic regions in the regulation of expression of their transcripts (Fig. 3H). Furthermore, in combination with the chromatin immunoprecipitation sequencing (ChIP-seq) data from the literature, we found that several of these peaks in the cytotoxicity-related genes could be bound by the TFs of CTL relevance [eomesodermin (*EOMES*), T-bet, and FOS-like antigen 2 (FOSL2)] (Fig. 3H) (*40*–*42*). We then assessed the motif enrichment across these clusters and found that these CTL-specific TFs showed higher motif enrichment in cluster 6 followed by cluster 7, while the naïve/long-term memory–specific TFs, lymphoid enhancer-binding factor 1 (LEF1) and TCF1, showed no major noticeable difference (Fig. 3I and fig. S3E). We also noted that the cluster 6 cells were predicted to have cells from both clusters 11 and 12 of scRNA-seq data (clusters 6-11 and 6-12), indicating further heterogeneity at the level of chromatin accessibility (Fig. 3G). Genomic tracks for cytotoxicity-associated genes confirm this variability within cluster 6, where clusters 6-11 and 6-12 show differential accessibility for some genes such as *GZMB*, *SLAMF7*, and *PRF1*, while other genes such as *CCL5* and *TBX21* showed no variation (Fig. 3H). Together, these observations suggest a tight regulation of the gene expression of cytotoxicity-related genes in T_SCM-CTL_. Overall, scRNA-seq and scATAC-seq suggest heterogeneity in the T_SCM_ subset and may indicate committed stem-like cells to different T cell lineages, where the T_SCM-CTL_ cells are poised for the CTL lineage. To understand whether the T_SCM-CTL_ cells are likely the close parallels to the most recently described TCF1^+^PD1^+^TOX^+^ stem-like precursors of exhausted T cells and the correlates of protection described in mouse models of both chronic and acute viral infection, we examined the expression of thymocyte selection–associated high-mobility group box (TOX) and programmed cell death 1 (PD1) in T_SCM-CTL_ cells (*43*, *44*). We noted that the T_SCM-CTL_ cells within the T_SCM_ subset (marked by the expression of GPR56) coexpressed TOX and PD1, thus indicating that they closely resemble the TCF1^+^PD1^+^TOX^+^ stem-like precursors described in mouse (Fig. 3, B, C, and J, and fig. S3I).

Next, to identify TFs of unknown CTL function, which could play a role in early commitment to the CTL lineage, we examined the transcript expression of all the known TFs (1639) found in the T_SCM_ single-cell transcriptome dataset (1163 of the 1639 known TFs in the human genome) (*45*, *46*). The hierarchical clustering based on the expression of these 1163 TFs identified cluster 5 closely resembling the T_SCM-CTL_ clusters 11 and 12 (fig. S3F). To identify the potential TF of CTL relevance among them, we identified TFs that showed shared expression between clusters 5, 11, and 12 by performing differential expression analysis between these clusters versus the rest of the clusters (fig. S3G and data file S4). Of particular interest was a set of 29 TFs that included *STAT4*, *NFAT5*, *KLF12*, *NFATC3*, etc., that were coexpressed by cluster 5 and the T_SCM-CTL_ clusters 11 and 12, with cluster 12 showing relatively higher expression than cluster 11 (fig. S3, G and H). Among these, some are already reported to have a function in effectors [nuclear factor of activated T cells 5 (NFAT5) and signal transducer and activator of transcription 4 (STAT4)] or known for their role in stemness or proliferation (TCF12 and KLF12) (*30*, *47*–*49*). These observations suggest that several of these TFs may potentially regulate the CTL program, although further experimental validations are necessary to prove or disprove their role in commitment to the CTL program. Hence, cluster 5 may be the pre-T_SCM-CTL_ cluster from which the T_SCM-CTL_ cells develop. Overall, the cells in clusters 5, 12, and 11 are the progenitors that chronologically appear during the development.

### The CD4-CTLs are developed from T_SCM-CTL_ cells

**Fig. 4.**
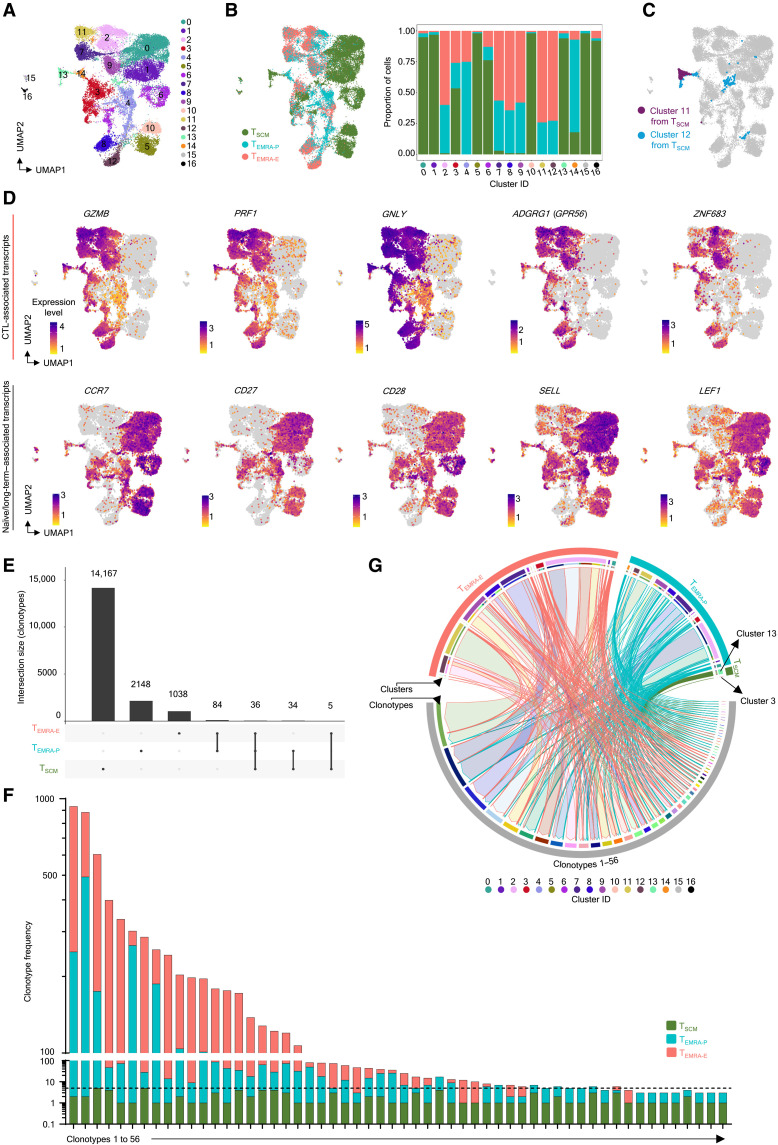
CD4-T_SCM-CTL_ cells are progenitors of CD4-T_EMRA_. (**A** and **B**) Integrated 2D UMAP embedding of scRNA-seq analysis of T_SCM_ (17,364 cells) and T_EMRA_ (15,677 cells) shown on the basis of either clusters (A) or origin [(B), left]. The stacked bar graph [(B), right] shows the proportion of cells from each category across different clusters (*x* axis). (**C**) 2D UMAP visualization of cells from clusters 11 and 12 from scRNA-seq analysis of T_SCM_ (T_SCM-CTL_ cells) in the integrated data. (**D**) 2D UMAP visualization of normalized expressions of indicated transcripts from specified categories. (**E**) The UpSet plot shows the frequency (*y* axis) and distribution of unique or shared clonotypes (connected lines) across categories. (**F**) The stacked bar plot shows the frequency and distribution of shared clonotypes between the indicated subsets. (**G**) The Circos plot shows distribution across clusters and categories (T_SCM_, T_EMRA-P_, and T_EMRA-E_) of 56 shared clonotypes between T_SCM_ and T_EMRA_ cells. The links between the corresponding cells with their clonotypes are colored on the basis of the clonotype with the arrowhead pointing toward the clonotype and the border of the link colored on the basis of the origin of the cell.

We then examined whether T_SCM-CTL_ cells are the precursors of the CD4-CTL lineage by comparing the single-cell transcriptomes and single-cell TCR (scTCR) repertoire of CD4-T_EMRA_ and CD4-T_SCM_ subsets from the same donors. The scRNA-seq analysis showed that most of the clusters were either T_SCM_-specific or T_EMRA_-specific, separating on the basis of longevity and effector status (Fig. 4, A and B, and fig. S4A). The T_SCM-CTL_ clusters (Fig. 3, A to D, clusters T_SCM_-C11 and T_SCM_-C12) mostly mapped back to (integrated T_SCM_ and T_EMRA_ data; iT_SCM-RA_) clusters iT_SCM-RA_-C13 and iT_SCM-RA_-C14 and part of iT_SCM-RA_-C3 (Fig. 4C). This observation was further validated by the expression of CTL-associated (*GZMB*, *PRF1*, *GNLY*, *GPR56* and *ZNF683*) and naïve/long-term memory–associated (*CCR7*, *CD27*, *CD28*, *SELL*, and *LEF1*) transcripts (Fig. 4D). We then analyzed the scTCR-seq data to establish developmental lineage connection. While ~76% of the T_EMRA_ cells were clonally expanded, which were distributed across multiple clusters and different clonotype expansion groups, only 1.23% of the T_SCM_ cells were clonally expanded and more than 55% of these expanded cells were found in iT_SCM-RA_-C13, the cluster dominated by the T_SCM-CTL_ cells (Fig. 4, B and C; fig. S4B; and data file S5). Furthermore, we noted that, of the 76% expanded T_EMRA_ cells, 67.5% of the cells shared clonotypes with the T_SCM_ subset, which were predominantly found in iT_SCM-RA_-C13 (99%), the T_SCM-CTL_ cluster (fig. S4B and data file S5). In a combined TCR clonotype analysis, we noted as many as 75 unique TCR clonotypes shared between T_EMRA_ and T_SCM_ subsets, of which 56 were clonally expanded in the T_EMRA_ subset (Fig. 4, E to G, and data file S5). The TCR clonotype sharing between T_SCM_ and T_EMRA_ was observed in 100% of the donors (fig. S4, C and D, and data file S5). These clonotypes themselves were predominantly not expanded in T_SCM_, with their frequency ranging from only 1 to 6; however, these clonotypes were hugely expanded in the T_EMRA_ subset with few clonotype frequencies being as high as 931, 883, and 600 (Fig. 4, F and G; fig. S4D; and data file S5). The majority of these shared clonotypes were found in cluster iT_SCM-RA_-C13 cells that carry T_SCM-CTL_ cells across all the donors (Fig. 4G, fig. S4D, and data file S5). Overall, overlaying the single-cell transcriptomic data with the paired scTCR repertoire data, we found a previously unknown subset in the T_SCM_ compartment—the T_SCM-CTL_, the long-term memory subset with stemness properties—that is likely poised to differentiate to the CD4-CTL lineage in an immunological event such as infection.

### Differentiation of CD4 T_N_ cells to the CD4-CTL phenotype

**Fig. 5.**
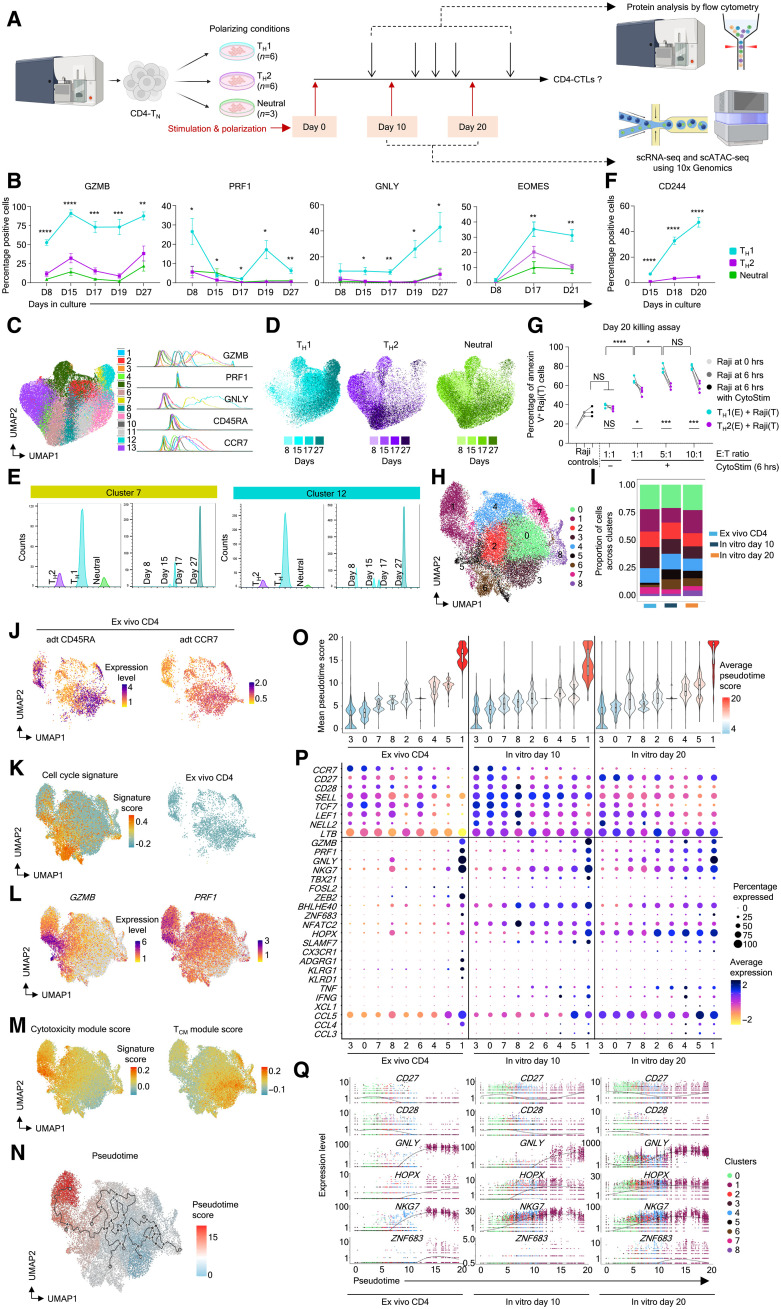
In vitro differentiation and polarization of naïve CD4 T (T_N_) cells to CD4-CTLs. (**A**) Schematic representation of experimental design. Created in BioRender. V. S. Patil (2025); https://biorender.com/a95zbu0. (**B**) Summary flow cytometry data of indicated proteins across days. (**C** to **E**) Integrated 2D UMAP visualization of multifactorial protein analysis of 132,582 in vitro–differentiated and T_H_1, T_H_2, and neutral polarized cells. Clusters [(C), left] are visualized by their geometric mean fluorescence intensity for the indicated proteins [(C), right]. The UMAP (D) and histogram (E) are split by polarization and colored by days. (**F**) Expression of CD244 across indicated conditions and days. T_H_1 (*n* = 6), T_H_2 (*n* = 6), and neutral (*n* = 3) [(B) and (F)]. (**G**) The matched scatterplot shows the percentage of AV^+^ cells in the indicated groups (*n* = 4). hrs, hours. (**H**) Integrated 2D UMAP embedding of single-cell transcriptomes of 26,815 cells from ex vivo CD4 T cells and in vitro–differentiated and T_H_1 polarized T cells from D10 and D20, colored by clusters. (**I**) The stacked bar graph shows the distribution of cells across clusters in each indicated category. (**J**) 2D UMAP visualization of indicated normalized protein expression. (**K**) 2D UMAP projection painted by cell cycle gene signatures either together (left) or for ex vivo CD4 data (right). (**L** to **N**) 2D UMAP visualization painted by normalized expressions of indicated transcripts (L) or by indicated gene signatures (M) or by pseudotime scores (N). (**O**) Violin plot for the mean pseudotime score across clusters painted by the average score. (**P**) The dot plot shows the mean expression and percentage of expressing cell of indicated transcripts. (**Q**) Scatterplot of normalized expression of indicated transcripts (*y* axis) along the pseudotime trajectory (scored along the *x* axis) colored on the basis of clusters. Error bars [(B), (F), and (G)] represent the means ± SEM. NS, *P >* 0.05; *P < 0.05; **P < 0.01; ***P < 0.005; and ****P < 0.001 from Student’s paired two-tailed *t* test [(B), (F) (T_H_1 versus T_H_2), and (G)].

The molecular program that drives the naïve T_H_ cells to the cytotoxic (CTL) lineage in humans is poorly understood. Hence, to systematically understand the developmental lineage of CD4-CTLs in humans, we wanted to develop an in vitro differentiation model to generate CD4-CTLs with cytotoxic potential from CD4-T_N_ cells and track them over a period. Considering few overlapping gene expression patterns between CD4-CTLs and T_H_1 cells, we hypothesized that the stimulation of T_N_ cells with T_H_1-specific polarizing milieu of cytokines and blocking antibodies of other T_H_ lineages, over a period, may differentiate them to CD4-CTLs. Hence, to test this hypothesis, we activated the T_N_ cells via TCR stimulation using αCD3/αCD28 beads for 48 hours and continued to culture them in a cocktail of T_H_1 or as controls in T_H_2 and neutral polarizing cytokines and blocking antibodies along with IL-2 for 8 days (2 + 8–day cycle) (Fig. 5A). The successful polarization of T_N_ cells to the T_H_1 or T_H_2 lineage was confirmed by the expression of cytokines and TFs of their respective linages: IFN-γ and T-bet for T_H_1 and IL-4 and GATA3 for T_H_2 (fig. S5A). In alignment with our hypothesis, we observed a higher proportion of cells making cytolytic molecules such as GZMB, PRF1, and GNLY and CTL-TF EOMES under T_H_1 compared to T_H_2 or neutral polarizing conditions (Fig. 5B). Consistent with this observation, previous reports in both human and animal models have also shown that the T_H_1 polarizing cytokines can induce the expression of GZMB and PRF1 (*50*–*52*). The expression of these cytotoxicity-associated markers was dynamic and was more pronounced from the second round of stimulation [day 15 (D15)/D17 onward], indicating that constant exposure to desirable cytokine milieu can polarize naïve T_H_ cells to T_H_ cells expressing cytolytic molecules (CD4-CTLs) (Fig. 5B). We then performed an integrated multifactorial analysis that included the expression of cytolytic molecules, GZMB, PRF1, and GNLY, and memory markers CD45RA and CCR7; different polarizing conditions; days after polarization; and donors (*n* = 3 to 6) to understand the dynamic changes during the differentiation process using the flow cytometry data (Fig. 5, C to E, and fig. S5B). Unbiased clustering using a similar number of cells from T_H_1 and T_H_2 revealed that they were represented by distinct clusters, and the ones (7 and 12) showing a relatively higher expression of the cytolytic molecules GZMB, PRF1, and GNLY predominantly belonged to the T_H_1 group (Fig. 5, C to E, and fig. S5B). Further within the T_H_1 group, there was a progressive increase with days (D8 to D27) in the cells coexpressing cytolytic markers GZMB, PRF1, and GNLY that also showed a higher expression of CD45RA and a lower expression of CCR7, hence suggesting that under the T_H_1 polarizing condition, the naïve T_H_ cells gradually acquire the expression of cytolytic molecules and appear to resemble CD4-T_EMRA_ cells (Fig. 5, D and E). The examination of other CTL-associated markers such as CD244, KLRG1, and CX3CR1 revealed dynamicity in marker expression—while the CD244 expression gradually increased from D15 to D20 (average of 6.83 to 47.28%), the expression of KLRG1 and CX3CR1 was undetectable under the T_H_1 polarized condition, whereas no or very few cells expressed any of the three markers under T_H_2 or neutral polarizing conditions (Fig. 5F and fig. S5C). Furthermore, in an in vitro functional cytolysis assay, where the target cells (B lymphoblastic cell line, Raji) and T cells were engaged using a superantigen (CytoStim, Miltenyi Biotec), the cells polarized under T_H_1 conditions showed higher percentages of target apoptotic cells [annexin V–positive (AV^+^)] compared to T_H_2 polarized cells across all E:T (effector:target) ratios on D15 and D20 of polarization (Fig. 5G and fig. S5, D and E). We also noted that the cytolysis of the target cells by the T_H_1 polarized cells was predominantly TCR-dependent (Fig. 5G). Together, these results show that naïve T_H_ cells can be differentiated to T_H_-CTL (CD4-CTL)–like cells under the T_H_1 polarizing condition over a continued culture in a polarizing milieu and suggest a dynamic and gradual expression of cytolytic molecules during the development of the CTL program.

Given that we observed a significant increase in the expression of cytolytic molecules and cytolysis of target cells under the T_H_1 polarizing condition compared to the T_H_2 or neutral condition, further to understand the overall cellular heterogeneity and the dynamic changes in the molecular program of CTL development, we performed paired scRNA-seq and scTCR-seq on naïve cells polarized under the T_H_1 condition at two different stages: D10 (potentially committed to the CTL lineage) and D20 (cells with the active CTL program) (Fig. 5A). To understand the developmental trajectories of these in vitro–differentiated cells in the context of well-defined developmental memory stages, we compared them with the single-cell transcriptomes of ex vivo–isolated, unstimulated total CD4^+^ T cells from the same donors (Fig. 5H and fig. S5F). The integrated scRNA-seq analysis of 26,815 cells identified nine clusters revealing heterogeneity in differentiated cells that coclustered with ex vivo CD4^+^ T cell subsets classified on the basis of CD45RA and CCR7 as naïve (majorly cells from clusters 3 and 0 and part of 7 and 8), memory (T_CM_ + T_EM_), and T_EMRA_ (cluster 1) subsets (Fig. 5, H to J, and fig. S5, F and G). Most clusters, except clusters 5 and 6, consisted of cells from all groups, ex vivo CD4^+^ T cells, and D10 and D20 cells, indicating that the cells differentiated under the T_H_1 polarizing condition consist of cells that resemble different developmental ex vivo memory subsets (Fig. 5, H and I, and fig. S5, F and G). Clusters 5 and 6 comprised majorly in vitro–differentiated cells and were enriched for cell cycle signatures, hence justifying their absence in the quiescent ex vivo CD4 group (Fig. 5, H to K, and data file S2) (*53*). Cluster 1, which showed a higher expression of CTL-associated transcripts *GZMB* and *PRF1*, included CD4-T_EMRA_ cells (CD45RA^+^CCR7^−^) from the ex vivo CD4 group (Fig. 5, J and L). This was further confirmed by the enrichment of signature score for cytotoxicity/T_EMRA_ in cluster 1 that showed lower enrichment for long-term memory (T_CM_)/naïve gene sets (Fig. 5M, fig. S5H, and data file S2) (*9*). To further understand their developmental trajectories in an unbiased way, we performed pseudotime analysis using Monocle3 (Fig. 5N) (*54*). Overlaying the pseudotime scores, we could assign clusters to different developmental trajectories, with clusters 3 and 0 being the starting point with a naïve-like phenotype, cluster 1 being the end point with a T_EMRA_-like phenotype, and the rest of the clusters showing a continuity or transitioning states in memory subsets (Fig. 5O). Although cluster 1 expressed cytolytic molecules across all three groups (ex vivo CD4 T cells and in vitro–differentiated cells from D10 and D20), the in vitro–differentiated cells from other clusters also expressed cytolytic molecules, albeit at a reduced level, while the ex vivo CD4 group did not (Fig. 5P). Furthermore, when we assessed genes on the pseudotimescale, the naïve/long-term memory–associated genes (*CD28* and *CD27*) and effector-associated genes (*GZMB*, *PRF1*, and *GNLY*) were expressed by distinct clusters from the ex vivo CD4 group, while the cells from in vitro D10 or D20 groups coexpressed both sets of genes (Fig. 5, P and Q). The CTL-associated TFs showed dynamic expression patterns during the differentiation process; while *TBX21*, *FOSL2*, *BHLHE40*, and *NFATC2* showed a relatively higher expression at D10 compared to D20, especially in clusters positioned late in the pseudotimescales (clusters 2, 6, 4, 5, and 1), *HOPX*, *ZEB2*, and *ZNF683* showed a higher expression at D20 compared to D10 (Fig. 5, P and Q, and fig. S5I). We further noted that among the clusters of early pseudotimescale (3, 0, 7, and 8), cluster 8 showed a relatively higher expression of a few CTL/effector–associated transcripts such as *GNLY*, *NKG7*, *ZEB2*, *BHLHE40*, *NFATC2*, and *SLAMF7*. This prompted us to examine whether cluster 8 resembles the T_SCM_-C5 cluster (pre-T_SCM-CTL_; Fig. 3) and the development of CTLs from T_N_ cells is via pre-T_SCM-CTL_ cells. To this end, we observed that cluster 8 is enriched for the pre-T_SCM-CTL_ (T_SCM_-C5 cluster; Fig. 3 and fig. S3)–associated gene set when compared across the groups (fig. S5, J and K, and data file S4). Consistent with this observation, we further noted a higher expression of transcripts associated with TFs (*STAT4*, *NFAT5*, *KLF12*, *TCF12*, etc.) that we predicted to be potentially functioning upstream of CTL-TFs and the CTL program (figs. S3, G and H, and S5L). These results provide further evidence for CD4-CTL development from T_SCM-CTL_ cells that are mostly developed from pre-T_SCM-CTL_ cells poised for the CTL program.

### The T_N_ cells polarized under T_H_1 undergo a gradual change to acquire the cytotoxicity program

**Fig. 6.**
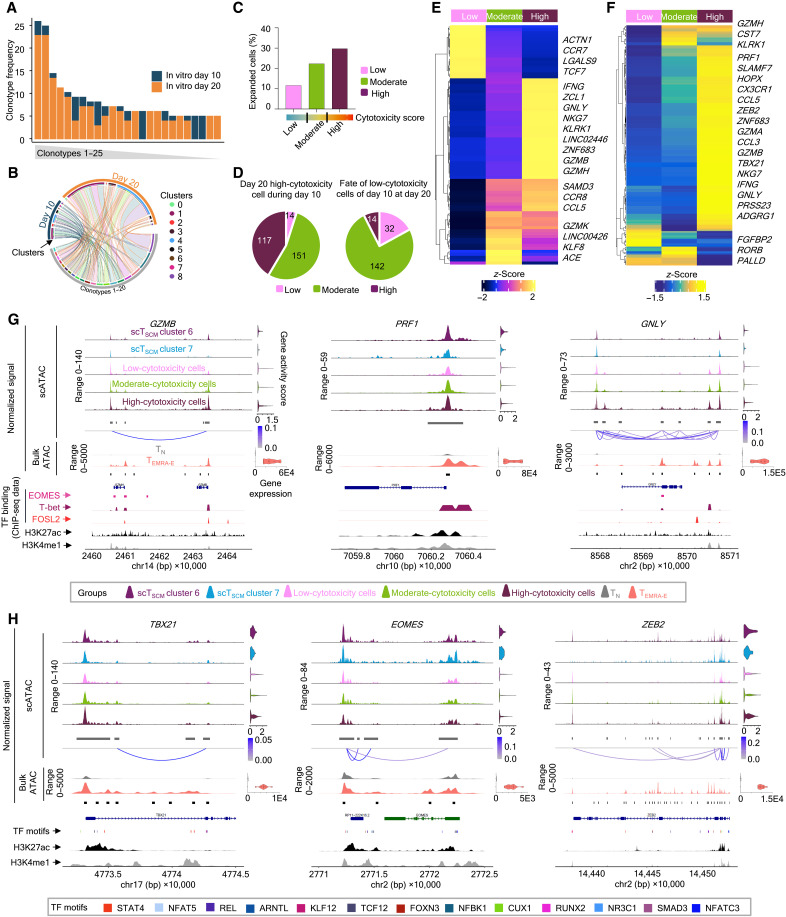
CD4-T_N_ cells polarized under T_H_1 conditions undergo a gradual change to acquire the cytotoxicity program. (**A**) The stacked bar graph shows the frequency of top 25 most expanded clonotypes across days. (**B**) The Circos plot shows top 20 shared clonotypes between D10 and D20 cells across clusters. (**C**) Bar graph of a proportion of expanded TCR (≥2) clonotypes across cytotoxicity signature–based categorized cells. (**D**) The pie chart shows the distribution of clonotypes grouped by cytotoxicity from the indicated time points. (**E**) The heatmap of scRNA-seq analysis shows the row-wise *z*-score of hierarchically clustered normalized expression of the 50 most significantly enriched transcripts from each category on the basis of the Wilcoxon rank-sum test. (**F**) The heatmap of scATAC-seq analysis shows the row-wise *z*-score of hierarchically clustered normalized gene activity (gene scores) of top 200 T_EMRA-E_ enriched transcripts in each category. For both (E) and (F), each column represents the average expression of all cells for a given category. (**G** and **H**) Single-cell (T_SCM_ and differentiated cells) or bulk (CD4-T_N_ and T_EMRA-E_, *n* = 10, aggregated) ATAC-seq analysis shows the chromatin accessibility of the indicated gene-containing genomic loci, visualized using CoveragePlot with a cluster-wise group gene score for single-cell open chromatin data or normalized gene expression (transcriptomic data) for bulk (T_N_ and T_EMRA-E_) data as violin plots (right) and significant coaccessible peaks connected via links colored on the basis of scores (graded blue scale, cutoff <0.05). ENCODE-sourced histone modification marks of H3K27ac and H3K4me1 are shown. Publicly available ChIP-seq data for T-bet, EOMES, and FOSL2 for the corresponding genomic loci shown as colored peak plots (G). TF of interest (different colors) having consensus motifs in peaks from scATAC data of in vitro–differentiated cells represented as a peak plot (H).

To understand the developmental trajectory of these cells, we analyzed the scTCR-seq data from the in vitro–differentiated cells. The TCR-seq analysis revealed sharing of clonotypes between D10 and D20, with 14 of the 25 most-expanded clonotypes being shared, with substantially higher expansion at D20 compared to D10 (Fig. 6A and data file S5). When the top 20 shared clonotypes were analyzed from both D10 and D20 cells across the clusters, clonotypes found in clusters 1 and 4 that are positioned later in the pseudotimescale showed further expansion at D20 from D10 (Fig. 6B and data file S5). We then classified these cells on the basis of the cytotoxicity signature score as low-, moderate-, and high-cytotoxicity cells to examine their trajectory of CTL program development (Fig. 6C and data file S2) (*9*). We noted a gradual increase in the proportion of clonally expanded cells from low-cytotoxicity to moderate-cytotoxicity to high-cytotoxicity cells (Fig. 6C and data file S5). Together, the scTCR-seq and scRNA-seq data suggest that the high-cytotoxicity cells at D20 are a close representation of ex vivo T_EMRA_ (CD4-CTL effector) cells. Thus, to delineate the path of cytotoxicity program development, we examined the TCR clonotypes found in high-cytotoxicity cells at D20 across the cells from D10 categories. We observed that although most cells from the high-cytotoxicity group at D20 are found in the high- or moderate-cytotoxicity group at D10, a few clonotypes were also found in the low-cytotoxicity group at D10 (Fig. 6D and data file S5). In a reverse analysis, we examined the fate of the low-cytotoxicity cells at D10 during D20 to understand whether they develop in a linear fashion. Most clonotypes observed in low-cytotoxicity cells at D10 were found in the moderate-cytotoxicity group, with minor amounts in the high-cytotoxicity group in D20 cells (Fig. 6D and data file S5). However, we also found a notable proportion of cells (more than 17%; 32 of 188) still remaining in the low-cytotoxicity group even at D20, potentially suggesting unequal cell division, ensuring that a small fraction of cells remains less differentiated, maintaining stemness, hence mimicking the CD4-CTL memory development ex vivo (Fig. 6D). Together, these observations suggest linear development for the CTL program as well as provide evidence for unequal division where two daughter cells can have different phenotypes because of nonequal division. Paralleling this observation, we further noted that the moderate-cytotoxicity cells share gene expression patterns of both low- and high-cytotoxicity cells at an intermediate level (Fig. 6E and data file S6). The high-cytotoxicity cells show a higher expression of CTL-associated cytokines and chemokines (*IFNG*, *CCL4*, *CCL5*, etc.) along with effector molecules (*GZMB*, *NKG7*, *KLRK1*, *GNLY*, etc.) (Fig. 6E and data file S6).

The open chromatin landscape can aid in dissecting the regulation patterns of gene expression. Hence, we performed scATAC-seq on cells polarized under T_H_1 conditions at D10 and D20. Similar to the scRNA-seq data, we could categorize cells as low-, moderate-, and high-cytotoxicity groups on the basis of the cytotoxicity module score for peaks in scATAC-seq data (Fig. 6F and data file S2). The gene activity of the T_EMRA-E_-enriched genes (versus T_N_; data file S1) across these three categories indicated that a large set of genes showed the highest activity in the high-cytotoxicity group, followed by moderate- and low-cytotoxicity groups (Fig. 6F). Furthermore, the scATAC-seq data analysis revealed that the peaks at the regulatory regions as well as the gene body of the CTL-associated genes such as granzymes (*GZMB* and *GZMH*), perforin (*PRF1*), granulysin (*GNLY*), *SLAMF7*, and *CD244*; cytokines such as *IFNG*; and the TFs associated with the CTL program, *TBX21*, *ZEB2*, *FOSL2*, and *HOPX*, showed gradual opening from low-cytotoxicity to moderate-cytotoxicity to high-cytotoxicity cells; however, in few genes such as *EOMES* and *BHLHE40*, the variation between the categories was minimal, especially at the transcription start site (TSS) (Fig. 6, G and H, and fig. S6, A and B) (*9*, *14*, *32*–*34*). These groups also show the motif activity for TFs of both CTL relevance and long-term memory/naïve–associated in a graded fashion, with high-cytotoxicity cells showing the highest activity for T-bet, EOMES, and FOSL2 and the low-cytotoxicity ones showing relatively higher activity for TCF1 and LEF1 (fig. S6C). To understand the chromatin accessibility of these in vitro–differentiated CTLs, in the context of ex vivo cells, we then compared them with chromatin accessibility profiles of the T_SCM-CTL_ clusters (clusters scATAC-T_SCM_-C6 and scATAC-T_SCM_-C7; Fig. 3, E to G), as well as the CD4-T_N_ cells from which programming to the CTL lineage was initiated and the CD4-T_EMRA-E_ cells, the bona fide CD4-CTL effector cells (Fig. 2C), and further overlayed it with the histone activation marks such as H3K4me1 and H3K27ac from the ENCODE database (Fig. 6, G and H, and fig. S6, A and B) (*36*–*38*, *55*). The open peaks at the genomic loci of the CTL-associated genes in in vitro–differentiated cells matched with those of the CD4-T_EMRA-E_ subset and were mostly missing from the CD4-T_N_ subset, suggesting the naïve cells that were differentiated to CD4-CTLs in vitro have the CTL program activated (Fig. 6, G and H, and fig. S6, A and B). For most of the genes, the open chromatin patterns of the moderate-cytotoxicity group matched with those of T_SCM-CTL_ cluster 6 (scATAC-T_SCM_-C6), and those of the low-cytotoxicity group matched with those of cluster 7 (scATAC-T_SCM_-C7), although we noted few peak-specific variations; EOMES showed no major difference between categories, and for *HOPX*, T_SCM-CTL_ cluster 6 (scATAC-T_SCM_-C6) resemble the low-cytotoxicity group (Fig. 6, G and H, and fig. S6, A and B). Furthermore, the peaks at the TSS of CTL-associated genes *GZMB*, *PRF1*, *SLAMF7*, and *IFNG* and CTL-TFs *TBX21*, *EOMES*, *ZEB2*, *FOSL2*, and *BHLHE40* correspond to overall activation marks H3K27ac and H3K4me1, an activation mark associated with promoters and enhancers (Fig. 6, G and H, and fig. S6, A and B) (*36*, *37*, *55*). We then compared the binding of CTL-TFs such as T-bet, EOMES, and FOSL2 in these peaks using ChIP-seq data from the literature and found that many of these chromatin regions can be bound by one or more of these TFs (T-bet, EOMES, and FOSL2) (Fig. 6G and fig. S6A) (*40*–*42*). The coaccessibility analysis (cicero-generated links) of these peaks revealed no specific pattern on the basis of the position of these peaks with respect to the gene body (upstream, downstream, or intronic regions) for the coaccessibility, indicating a complex and distinct regulatory mechanism used by these cells in activating different genes in the CTL program (Fig. 6, G and H, and fig. S6, A and B). Furthermore, comparing the intensity of peaks between low-cytotoxicity cells and cells from T_SCM_ suggests that the in vitro–differentiated cells go through a phase where they represent the T_SCM-CTL_ cells. We also observed that the genomic region around the CTL-TFs had motifs for several of the previously unidentified TFs (13 of 29 where motif information was available) (Fig. 6H and fig S6B). Together, these observations suggest a possible regulation of the CTL-TFs via different TFs binding to different regulatory regions on the basis of the developmental stage under consideration and thus may regulate the overall CTL program by directly regulating either CTL-associated genes or the CTL-TFs (Fig. 6H and fig. S6B). However, these observations need further detailed validation using ChIP-seq.

### T_SCM_ cells polarized under the T_H_1 condition acquire cytotoxicity faster than naïve (T_N_) cells

**Fig. 7.**
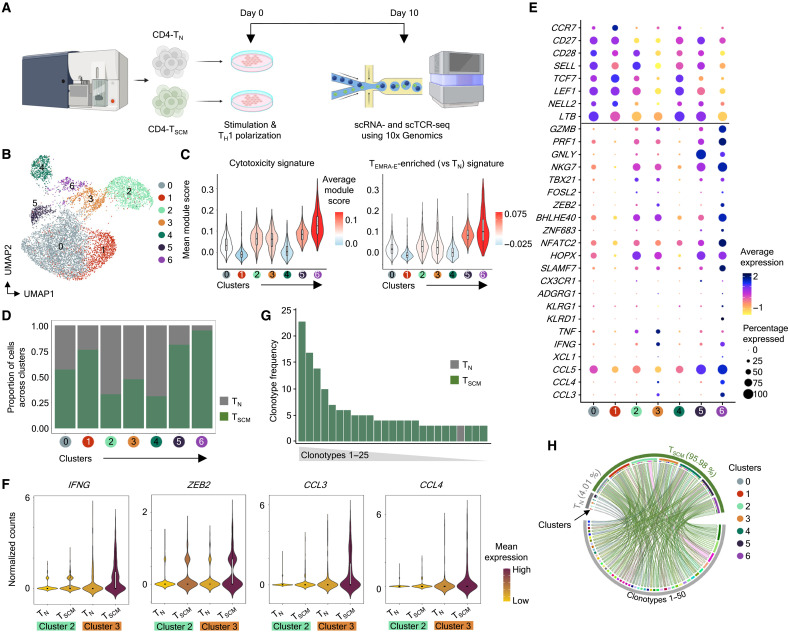
T_SCM_ cells acquire cytotoxicity faster than T_N_ cells under T_H_1 differentiation and polarization conditions. (**A**) Schematic representation of experimental design for the differentiation and polarization of human naïve CD4-T (T_N_) and CD4-T_SCM_ cells under the T_H_1 (*n* = 6) condition. Created in BioRender. V. S. Patil (2025); https://biorender.com/a95zbu0. (**B**) 2D UMAP projection of the scRNA-seq analysis of 8701 in vitro–differentiated and T_H_1 polarized naïve and T_SCM_ cells from D10. (**C**) Violin plot for the mean signature scores (*y* axis) for indicated gene modules per cluster painted by the average score. (**D**) Stacked bar graph of a proportion of cells from each origin across different clusters. (**E**) The dot plot shows the mean expression (color) and percentage of expressing cell (size) of indicated transcripts. (**F**) Violin plots show the mean expression across indicated clusters split between T_N_ and T_SCM_ for indicated transcripts. (**G**) The stacked bar graph shows the frequency of top 25 most expanded clonotypes, colored by origin. (**H**) The Circos plot shows top 50 clonotypes across T_N_ and T_SCM_ origin cells across clusters. Percentages in brackets indicate the contribution of each origin toward the top 50 clonotypes.

The analysis of ex vivo memory subsets has revealed that CD4-CTLs originate from T_SCM-CTL_ cells, so we hypothesized that if the in vitro differentiation is initiated from T_SCM_ cells, they are likely to achieve the CTL phenotype faster than the T_N_ cells. Thus, we parallelly differentiated and polarized the naive (T_N_) and T_SCM_ cells under the T_H_1 condition (Fig. 7A). The scRNA-seq analysis on D10 of the polarization revealed four cytotoxic clusters (clusters 2, 3, 5, and 6) with clusters 5 and 6 showing relatively higher module scores for cytotoxicity and effector gene sets (Fig. 7, B and C, and data files S1 and S2). These high cytotoxic clusters 5 and 6 contained a higher proportion of T_SCM_ cells compared to naïve cells (Fig. 7D). Both clusters 5 and 6 showed a higher expression of cytotoxicity-associated molecules such as *GZMB*, *GNLY*, *PRF1*, and *NKG7* and TFs such as *TBX21*, *BHLHE40*, *ZEB2*, *ZNF683*, etc. (Fig. 7E). Even within the other two cytotoxic clusters, 2 and 3, the T_SCM_ cells showed a higher expression of cytotoxicity-associated transcripts such as *IFNG*, *ZEB2*, *CCL3*, and *CCL4* compared to naïve cells, thus indicating that the T_SCM_ cells achieve the cytotoxic phenotype faster than naïve cells under T_H_1 polarizing conditions at D10 (Fig. 7F). The scTCR-seq analysis revealed that a relatively higher proportion of T_SCM_ cells is clonally expanded compared to naïve cells (Fig. 7, G and H, and data file S5). Of the top 25 most expanded clonotypes, 24 of them belonged to the T_SCM_ cells, accounting for more than 97% of the T_SCM_ cells expressing the top 25 clonotypes (Fig. 7G and data file S5). Even when we extended our analysis to the cells containing the top 50 expanded clonotypes, the T_SCM_ cells represented a higher proportion with the most expanded clonotypes predominantly observed in clusters 5 and 6 (Fig. 7H and data file S5). Overall, these results show that if the differentiation is started from the T_SCM_ subset, which contained precommitted CTL precursors, the CTL program is achieved faster than starting from the T_N_ cells.

## DISCUSSION

Although the CD4-CTLs have been identified in many diseases such as infectious diseases, a variety of cancers, autoimmune disorders, etc., in both human and animal models, their functional relevance has not been very well established mainly due to their rarity in the periphery and inaccessibility as a result of tissue residency in humans. Considering their protective nature against many infectious diseases, their proportions can be a valuable readout of correlates of protection in natural infection as well as upon vaccination and, hence, can be used to test the vaccine efficacy against these diseases. However, although CD4-CTLs have been shown to express many CTL-associated genes, they have not been studied in parallel to the classically known CTLs, the CD8-CTLs; hence, their functional relevance has remained questionable. By studying both of these CTLs parallelly from the same donors in similar settings, we have clearly shown that they are indistinguishable from each other with respect to their transcriptomic profile and TCR clonal expansion, as well as the cytotoxicity-associated protein expression (GZMB, PRF1, GNLY, CX3CR1, GPR56, etc.) was at par (Fig. 1). Hence, these results suggest that an exploration of both CD4-CTLs and CD8-CTLs’ induction during vaccination and cell-based therapies may provide better outcomes. These studies provide a goal for exploring the CD4-CTLs for therapeutic interventions, where the immune evasion by the pathogen happens to avoid CD8 T cell–mediated killing.

The lack of understanding of the developmental lineage of CD4-CTLs, unlike CD8-CTLs, has been a major hurdle in exploring their therapeutic value (*56*–*58*). Hence, in this study, our approach was to first identify a long-term memory subset that can develop into CD4-CTL effectors and memory in response to any immunological insult. Through an integrative analysis of multiomics approaches, we identified an extremely small subset within a rare memory compartment with stemness properties (T_SCM_) that is poised for the CD4-CTL lineage, the T_SCM-CTL_ subset (Figs. 2 and 3). TCR clonal sharing provided evidence for the development of CD4-CTLs from T_SCM-CTL_ (Fig. 4) (*9*). The dynamic nature of CTL-associated TFs may indicate that different stages of the CTL program can be regulated by different sets of TFs, and hence, they may modulate the expression of distinct groups of genes in the CTL program, with TFs such as Hobit (homolog of Blimp-1 in T cells) and HOPX (homeodomain-only protein homeobox) regulating the terminal differentiation of CTL effectors (Fig. 5, P and Q) (*9*, *14*, *26*, *32*). Furthermore, a deep dive into the heterogeneous T_SCM_ subset revealed a separate trajectory for CTL development with cells at different stages of development (T_SCM-CTL_ cells and pre-T_SCM-CTL_ clusters; T_SCM_-C5 → T_SCM_-C12 → T_SCM_-C11). These cells appear to be the close human CD4^+^ T cell counterparts of the most recently described precursors of CD8^+^ T cells that adapt to both chronic and acute infections and are the precursor of exhausted CD8^+^ T cells (*43*, *44*). Although not in the context of CD4-CTLs, similar cells have been described in CD4^+^ T cells in transplantation and cancer immunity in mouse models (*59*, *60*), thus establishing the potentially important role played by these stem-like cells in health and disease. Furthermore, we identified a set of 29 TFs expressed by pre-T_SCM-CTL_ cells and the effectors that may function upstream of the cytotoxicity program. Among these, some are already reported to have a function in effectors; NFAT5 has recently been shown to be associated with CD8 effectors and to induce exhaustion in CD8^+^ T cells and was also one of the differentially expressed genes (DEGs) associated with the CD4-T_EMRA-E_ subset (versus T_N_) in this study (data file S1), and STAT4 was the known regulator of T-bet in the T_H_1 lineage (*48*, *49*). However, many of these TFs have been shown to play a role in stemness or proliferation—TCF12, a member of the basic helix-loop-helix E (BHLHE) protein, has been shown to be expressed by many cell types including T and B cells and hematopoietic stem cells, and KLF12 is known to promote proliferation in cancer and natural killer cells (*30*, *47*). TCF12 is further known to form a heterodimer with other BHLHE proteins, which hence may form a complex with BHLHE40, a TF of known CTL relevance (*33*). These observations suggest that several of these TFs may potentially regulate the CTL program. However, knockdown or overexpression studies along with assays that can establish the occupancy of these TFs on the chromatin may provide confirmatory evidence for their role in the CTL program of both CD4^+^ and CD8^+^ T cells. Furthermore, experiments examining the paired open chromatin and transcriptome from the same cell at a single-cell resolution using tools such as the multiome (ATAC + RNA) from 10x Genomics can provide a better understanding of the concerted chromatin accessibility linked to gene expression.

One of the major roadblocks in exploring CD4-CTLs in clinical utilization has been their much smaller number compared to CD8-CTLs in the periphery, despite them being equally potent killers (*9*, *10*). This and other studies have demonstrated that naïve CD4 T cells can be differentiated and polarized in vitro to CD4^+^ T cells expressing CTL-associated genes such as GZMB, PRF1, GNLY, etc. (*52*). Furthermore, using single-cell multiomics, we show that one can generate CD4^+^ T cells with cytolytic potential in vitro that are at different developmental stages. Different categories within the in vitro–differentiated cells mimicked different ex vivo subsets; while some mimicked the T_EMRA-E_, others mimicked either the earlier or later stage of the stem-like memory precommitted to the CTL lineage, thus representing a variety of stages of CTL development. The in vitro–polarized CD4-CTLs, while maintaining the cytolytic ability, showed signatures of long-lived memory phenotype, thus overcoming one of the major challenges with ex vivo–isolated effectors that are unable to proliferate beyond a point because of anergy or exhaustion (*61*, *62*). Thus, the system developed here can be further explored for therapeutic applications. The generation of a long-lived memory subset, which has the potential to quickly differentiate to effectors, has always been considered an important goal in both natural infections and vaccinations to maintain long-term immunity against any pathogen so that, in the event of reinfection from the same pathogen, the long-term memory can quickly differentiate to effectors and provide protection against the invading pathogen. Overall, in this study, we show the potential of CD4-CTL effectors in direct comparison with CD8-CTL effectors and also delineated the developmental lineage of CD4-CTL from naïve to T_SCM-CTL_, a stem-like CD4-CTL memory subset, to CD4-CTL effectors. The data and the knowledge base generated in this study will pave the way for exploring the therapeutic potential of CD4-CTL effectors in a variety of diseases including infectious diseases, autoimmune disorders, and cancers.

## MATERIALS AND METHODS

### Study subjects

Buffy coat samples were obtained from healthy donors who donated blood in Safdarjung Blood Bank during the period from October 2019 to June 2024. The donors consented to the use of samples for research. Donors were HIV-negative and had no history of hepatitis C infection. The median age was 29 years (ranging from 19 to 50 years), and all these donors were male.

### Ethics approval statement

Approval for the use of human material for research was obtained from the institutional human ethics committees from both National Institute of Immunology, New Delhi (IHEC no. 119/19), and Vardhman Mahavir Medical College (VMMC) and Safdarjung Hospital (SJH), New Delhi, India (IEC/VMMC/SJH/Project/2019-08/82). The donors consented for the use of the samples for research.

### PBMC isolation

PBMCs were isolated from the buffy coats by density gradient centrifugation using Ficoll-Paque Premium (GE Healthcare Biosciences). PBMCs were cryopreserved in 90% fetal bovine serum (FBS) supplemented with 10% dimethyl sulfoxide.

### Flow cytometry

Cryopreserved PBMCs were thawed, blocked using human immunoglobulin G (1:25), stained using fluorescence-conjugated cell surface protein antibodies’ cocktails in magnetic-activated cell sorting (MACS) buffer [phosphate-buffered saline (PBS) supplemented with 2% FBS and 2 mM EDTA], and analyzed for protein expression using flow cytometry. For intracellular protein staining, surface-stained cells were washed and fixed using Cyto-Fast Fix/Perm Buffer Set (cytokines and other intracellular proteins) or True-Nuclear Transcription Factor Buffer Set (for TFs) from BioLegend, followed by staining with a cocktail of antibodies (table S1). The stained cells were analyzed using BD LSR Fortessa or BD Symphony A5. For studies involving degranulation and cytokine secretion assays, cryopreserved PBMCs were thawed and rested for 2 hours at 37°C with 5% CO_2_ before stimulation using CytoStim (Miltenyi Biotec) for 6 hours. The degranulation marker LAMP1 (CD107a) antibody was added during the stimulation, and brefeldin A was added after 2 hours of start of stimulation to stop vesicular export. After 6 hours of stimulation, the cells were processed for analysis using flow cytometry. All flow cytometry data were analyzed using BD FlowJo version 10.8.1, and the geometric mean fluorescence intensity along with population percentages was exported and visualized using GraphPad Prism version 10.2.2.

T cell populations of interest were sorted from PBMCs after surface staining using BD FACS Aria Fusion in collection media (1:1 FBS:PBS). For cell culture, the cells were washed and cultured as per the experimental conditions. For downstream genomics experiments, the cells were collected in collection media supplemented with a 1:100 recombinant RNase inhibitor (RRI; Takara). For experiments involving bulk RNA-seq, the sorted cells were washed with 1× PBS before lysing using the TRIzol reagent (Invitrogen) and stored at −80°C for RNA extraction later.

### Bulk RNA-seq

The cryopreserved TRIzol samples were thawed and used for RNA isolation using the RNeasy Micro kit (Qiagen) as per the manufacturer’s recommendations. Bulk RNA-seq was performed as described previously using the Smart-Seq2 method (*9*, *63*) with 3 ng of total RNA. Briefly, the RNA was reverse transcribed, and cDNA was preamplified for 15 cycles followed by size selection using 0.8× Ampure XP beads (Beckmann Coulter). Quality control was performed using Fragment Analyzer 5200 (Agilent) and quantified using the Quant-iT PicoGreen dsDNA Assay kit (Invitrogen). Final libraries were prepared using the Nextera XT DNA Library Prep kit (Illumina, no. FC-131-1096) following the manufacturer’s protocol using 1.5 ng of preamplified cDNA. Libraries were then pooled and sequenced on NovaSeq 6000 (Illumina) for 150–base pair (bp) paired-end sequencing.

### Bulk RNA-seq data analysis

Raw demultiplexed FASTQ files (*n* = 70; 10 donors, seven cell types) were adaptor trimmed using trim_galore (https://github.com/FelixKrueger/TrimGalore) and mapped to the GRCh38 (hg38) human reference genome using STAR. Quality control was performed using fastqc, and count files were generated using HTSeq (*64*). DESeq2 was applied on depth-normalized gene counts obtained from HTSeq using design = “~0 + condition + donor” (*65*). Genes with counts <100 across all samples together were removed from downstream analysis. Pairwise cell type comparisons were performed, and genes were considered differentially expressed if it satisfied the parameters Benjamini-Hochberg–adjusted *P* ≤ 0.05 and ≥2 fold change. Annotations for gene biotype were imported from EnsDb.Hsapiens.v86.

#### Visualization

PCA plots were generated on most variable genes from variance-stabilization-transformation (vst)–applied data (Fig. 1F and fig. S1D) or based on the CD4-T_EMRA_–enriched (versus T_N_) gene list (fig. S2A and data file S1). Heatmaps were generated using the ComplexHeatmap package with cell type and donor information added as part of top_annotation to maintain uniformity across heatmaps (*66*). The EnhancedVolcano (https://github.com/kevinblighe/EnhancedVolcano) package was used for generating volcano plots of differentially expressed genes between cell types. Violin plots to represent normalized counts across cell types were generated using GraphPad Prism version 10.2.2.

#### *Gene set enrichment analysis* (*GSEA*)

GSEA was used to assess whether specific gene signatures were significantly enriched between two groups, as previously described (*21*). ClusterProfiler was used to perform GSEA between two cell types using ranked genes from DESeq2 comparisons (data file S1), and gseaplot2 from the enrichplot package was used to visualize the GSEA plot (*67*). The gene sets from the literature used for GSEA are mentioned in data file S2.

### Bulk TCR-seq

Bulk TCR-seq was performed as described previously (*9*, *25*). Briefly, 5 ng of total RNA (samples from the same RNA isolation used for bulk RNA-seq) was initially reverse transcribed using SMARTScribe Reverse Transcriptase (Takara) supplemented with a template switch oligo for TCR-seq along with TCRα and TCRβ gene-specific barcoded primers (*9*, *25*), followed by purification using 1× Ampure XP beads. The purified cDNA was then amplified for the TCRα and TCRβ regions using a nested polymerase chain reaction (PCR). The first PCR amplification was done for 20 cycles using Q5 Polymerase (NEB) followed by purification using 1× Ampure XP beads. The reaction volume was then split into two parts, and TCRα and TCRβ were amplified separately in a second nested PCR amplification step. Postamplification, the purified, sample-barcoded DNA was quantified using the Quant-iT PicoGreen dsDNA Assay kit (Invitrogen), and 500 ng to 1 μg of each were pooled for final library preparation using the NEBNext Ultra II DNA Library Prep Kit for Illumina using the manufacturer’s recommendations. Pooled libraries were sequenced using NovaSeq 6000 (Illumina) for 150-bp paired-end sequencing.

### Bulk TCR-seq data analysis

Raw FASTQ files were demultiplexed, mapped, and analyzed using MIGEC software with default settings (*24*, *25*). For clonotype analysis, the V segment, followed by the entire CDR3 region (nt), and the J segment were considered for TCRα, while for TCRβ, in addition, the D segment was also considered, with the counts taken from the output of *FilterCdrBlastResults* of the MIGEC pipeline (*25*). From VDJtools, the *caldiversitystats* package was used for calculating the Shannon-Weiner diversity index, while *calcsegmentusage* was used to calculate the usage of V and J segments across samples (*24*). A clonotype was considered expanded if the frequency of occurrence was ≥3 in the individual sample (data file S3).

### Bulk ATAC-seq

Largely, the Omni ATAC-seq protocol was followed with slight modifications (*68*). Cells sorted in collection media (1:1 FBS:PBS) were counted, and 55,000 cells were taken in LoBind 0.6-ml microcentrifuge tubes (Eppendorf) and washed with 0.5 ml of chilled PBS. The cell pellet was resuspended in 50 μl of chilled freshly made lysis buffer (10 mM tris-HCl, pH 7.4, 10 mM NaCl, 3 mM MgCl_2_, 0.1% IGEPAL-CA630, 0.1% Tween 20, 0.01% digitonin, 1× protease inhibitor, and 20 mM NaBu) supplemented with RRI (1 U/μl; Takara). After 5 min of lysis on ice, 0.2 ml of wash buffer (10 mM tris-HCl, pH 7.4, 10 mM NaCl, 3 mM MgCl_2_, and 0.1% Tween 20) supplemented with RRI (1 U/μl) was added and centrifuged. Pelleted nuclei were then transposed using Tn5 transposase from Illumina in a 50-μl reaction mix supplemented with 1× PBS, 0.1% Tween 20, and 0.01% digitonin at 37°C for 30 min. Tagmented DNA was purified using the Zymo DNA Clean and Concentrator-5 kit followed by amplification using barcoded Nextera XT primers with KAPA Hi-Fi HotStart Ready Mix. After the initial five cycles of amplification, cycle threshold determination (CTD) was performed using SYBR Green I nucleic acid gel stain (Invitrogen, no. S7563) and ROX dye (Invitrogen, no. 12223-012). On the basis of the CTD, additional cycles (CTD-2 cycle) were performed on the amplified DNA. Final libraries were purified and size selected using double-sided purification by Ampure XP beads (0.5×/1.8×). Quality control and quantification were performed using the Fragment Analyzer 5200 (Agilent) and Quant-iT PicoGreen dsDNA Assay kit (Invitrogen), respectively. Pooled libraries were sequenced using NovaSeq 6000 (Illumina) for 150-bp paired-end sequencing.

### Bulk ATAC-seq data analysis

Raw demultiplexed FASTQ files (*n* = 60; 10 donors, six cell types) were quality filtered and adaptor trimmed using trim_galore (https://github.com/FelixKrueger/TrimGalore), and trimmed reads were mapped to the GRCh38 (hg38) human reference sequence using Bowtie2 (*69*). Publicly available pipelines from snakemake (https://github.com/epigen/atacseq_pipeline) and nf-core were followed to analyze ATAC-seq data. Deduplicated reads [using picard (http://broadinstitute.github.io/picard) markduplicates] were filtered for reads mapping to the ENCODE-blacklisted region, mitochondrial genome, and low-quality reads (bedtools and samtools) (*70*, *71*). Reads passing all quality checks were called for peaks using MACS2 narrowPeak caller (*72*). Index-sorted and filtered BAM files, along with the narrowPeak called peak file, were used to generate the count matrix using diffBind with *summits=100*, and peaks were annotated using ChIPseeker (*73*) with the organism database “TxDb.Hsapiens.UCSC.hg38.knownGene” with *tssRegion* as −1000 to +100.

#### Visualization

PCA plots (fig. S2E) were generated using diffBind (http://bioconductor.org/packages/release/bioc/vignettes/DiffBind/inst/doc/DiffBind.pdf) from the normalized peak count matrix (*74*). Merged BAM files (across 10 donors per cell type, merged using samtools) were converted to BigWig files [using the University of California, Santa Cruz (UCSC)’s *bedGraphToBigWig*] for visualization using UCSC genome browser. GeneHancer regulatory elements (Double Elite) from UCSC corresponding to the given genomic loci are shown in the coverage plot to mark the putative regulatory elements (Fig. 2C). Layered H3K27ac marks for the corresponding genomic loci from ENCODE are shown as a peak (histogram) plot (Fig. 2C). The ComplexHeatmap (*66*) package was used to generate heatmaps corresponding to peak counts. To compare with scATAC-seq data, the bulk ATAC-seq BigWig files were imported into Signac and visualized using CoveragePlot (Fig. 6, G and H, and fig. S6, A and B) (*35*).

### In vitro differentiation of naïve and T_SCM_ CD4 T cells

Ex vivo–isolated CD4^+^ T_N_ or T_SCM_ cells from three to six donors were activated using αCD3/αCD28 DynaBeads Human T-Activator (Invitrogen, no. 11131D) for 48 hours and cultured for 8 more days in T cell media (Figs. 5A and 7A; Iscove’s modified Dulbecco’s medium supplemented with 5% FBS and 2% human serum) along with IL-2 cytokine (40 U/ml; BioLegend) and gentamicin antibiotic (50 μg/ml; Invitrogen) along with either T_H_1 (*n* = 6 donors), T_H_2 (*n* = 6 donors), or neutral (*n* = 3 donors) polarizing conditions [T_H_1 polarizing condition: IL-12 cytokine (5 ng/ml; R&D System) and IFN-γ cytokine (10 ng/ml; BD Biosciences) with αIL-17 monoclonal antibody (mAb) (10 μg/ml; eBio64CAP17; eBiosciences) and αIL-4 mAb (5 μg/ml; clone 3007; R&D System); T_H_2 polarizing condition: IL-4 cytokine (10 ng/ml; R&D System) with αIL-12 mAb (10 μg/ml; clone 24910; R&D System), αIL-17 mAb (10 μg/ml; eBio64CAP17; eBiosciences), and αIFN-γ mAb (10 μg/ml; clone NIB42; BD Biosciences). Cells were restimulated with αCD3/αCD28 DynaBeads when they stopped to proliferate (10 days: 2-day stimulation + 8-day culture). A total of three stimulation cycles were performed, and the culture was continued for a total of 30 days. Flow cytometry analysis for protein expression was carried out on indicated days. For the cytokine analysis, cells were activated using phorbol 12-myristate 13-acetate/ionomycin for a total of 4 hours with an addition of brefeldin A at 2 hours to stop vesicular export, followed by flow cytometry analysis. Single-cell transcriptomic (scRNA) and scATAC-seq analyses were performed on T_H_1 polarized cells on D10 and D20.

Integrated multifactorial analysis of flow cytometry data across multiple days was performed for the coexpression after concatenating all samples across days and polarizing conditions postdownsampling to exactly the same number of live- and singlet-gated cells across samples using BD FlowJo software (version 10.8.1). The concatenated file was subjected to uniform manifold approximation and projection (UMAP) reduction (arXiv: 1802.03426v3 [stat.ML]) of multiparameter protein data using default settings in FlowJo, and a *k*-nearest neighbors density estimation–based clustering algorithm was performed using XShift with default settings to identify clusters on the basis of protein coexpression (*75*). *z*-Score expression was mean centered across channel values, and graphs were exported from FlowJo as the geometric mean fluorescence intensity.

#### Killing assay

The target cell killing potential of the in vitro–differentiated cells was examined by coculturing the T_H_1 or T_H_2 polarized cells from D20 with Cell Trace Violet (CTV; Invitrogen)–stained and rested (2 hours) B lymphoblastic cell line Raji for 6 hours in a round-bottom 96-well plate in E:T ratios of 1:1, 5:1, or 10:1. The T cells were activated and engaged with the B cells using CytoStim (Miltenyi Biotec) that acts as a superantigen. After 6 hours of coculture, the cells were stained with AV (Invitrogen) and PI (propidium iodide) and analyzed using flow cytometry. The Raji cells were distinguished from T cells using CTV. The CTV^+^ Raji cells were further gated for AV^+^ and PI^+^ to identify the percentages of apoptotic (AV^+^) Raji cells, indicative of target cell killing (fig. S5D). The CTV-stained Raji cell before and after incubation (with or without CytoStim) served as controls for spontaneous death. The CTV-stained Raji cells incubated with T cells without the CytoStim served as the control to assess any TCR-independent killing.

### Single-cell transcriptomic and scTCR-seq analyses

Single-cell transcriptomic and TCR repertoire profiling was carried out using 10x Genomics 5′ Immune Profiling solutions. Briefly, depending on the experimental requirements, either cryopreserved PBMCs were thawed for ex vivo experiments or in vitro–differentiated cells were taken from culture and stained with hashtag oligo-conjugated antibodies (BioLegend) to assign donor identities, and cells of interest were sorted on the basis of experimental requirement after staining with fluorescence-conjugated cell surface protein antibodies. Ex vivo–sorted or in vitro–cultured cells were thoroughly washed with 1× PBS and maintained at a viability >95%. To analyze a limited repertoire of cell surface proteins, the cells were further stained with oligo-tagged cell surface protein antibodies (TotalSeq C antibodies, BioLegend) on the basis of experimental requirement. For scRNA-seq of CD4-T_EMRA_ (Fig. 4), T_EMRA-P_ and T_EMRA-E_ were sorted individually, followed by staining of T_EMRA-E_ with an oligo-conjugated αCD4 TotalSeq C antibody to be able to demarcate them in downstream analyses, and pooled in a 1:1 ratio. About 25,000 cells per lane were loaded onto Chromium NextGEM Chip G or Chip K depending on kit version 1.1 or 2, respectively. Manufacturers’ recommendations were followed to generate libraries of captured cells for transcriptome, TCR repertoire, and cell surface proteins. Libraries were sequenced on NovaSeq 6000 or NextSeq 2000 (Illumina) as per the manufacturer’s recommendations on the basis of the kit version or 150-bp paired-end sequencing.

### scRNA-seq analysis

Demultiplexed raw FASTQ files corresponding to gene expression, associated V(D)J, and cell surface protein libraries from each lane of 10x Genomics run were mapped to the human reference genome GRCh38 using the 10x Genomics’ cellranger (version 7.1.0) multipipeline. Individual 10x Genomics run was processed independently using the R-based Seurat package (version 5.0.1) before combining multiple datasets (*76*). Cells from different donors were demultiplexed on the basis of the normalized hashtag oligo count matrix using MULTIseqDemux with default parameters, and the same was used to detect and remove doublets from the datasets (*77*). Quality control parameters were determined on the basis of the distribution of gene count and unique molecular identifier (UMI) counts for each dataset, and low-quality cells were filtered out: ex vivo CD4 data—gene count and UMI count <500; in vitro–differentiated cell data—gene count <1500 >8000 (D10), <500 >7000 (D20), and <500 >7500 (naïve and T_SCM_ D10) and UMI count <3500 >45000 (D10), <1000 >40000 (D20), and <500 >30000 (naïve and T_SCM_ D10); ex vivo T_SCM_ and T_EMRA_ data—gene count <500 >3500 and UMI count <500 >17500. Cells were further filtered out on the basis of the percentage of mitochondrial reads: in vitro–differentiated cells <20% and ex vivo–sorted T cells <10%. Post–quality control, individual datasets were normalized using SCTransform with *vst.flavor “v2”* along with regressing out the percentage of counts belonging to mitochondrial genes or ribosomal genes (*78*). While analyzing a relatively homogeneous T cell population, to avoid the dominant effect of TCR genes for clustering analysis, all *TR*(*A/B/D/G*)(*V/J/C*) genes were removed from the transcriptome dataset before calculating principal components (PCs). A shared nearest-neighbor (SNN) graph was constructed using the *FindNeighbors* function using the most significant PCs determined empirically using *ElbowPlot*, and then clusters were determined using the *FindClusters* function using the SLM algorithm. The cluster resolution was chosen empirically across datasets using the clustree function on the principle that each cell cluster should express a unique group of genes. UMAP was applied on the basis of the above-described SNN graph to visualize the single-cell transcriptional profile in a two-dimensional (2D) space using the same number of most significant PCs. The removed TCR genes were added back to the count matrix before proceeding for downstream analyses. The cell surface protein data were normalized using the *NormalizeData* function with *normalization.method = “CLR”* (centered log ratio transformation). Briefly, for each cell, the hashtag counts were divided by the geometric mean of counts of all unique hashtags before log transformation.

#### Combining datasets

Experiments across different batches were integrated to mitigate batch effects using Seurat functions *FindIntegrationAnchors* and *IntegrateData* using 3000 most variable features selected across individually normalized datasets (*76*). An SNN graph was then calculated on the basis of the top PCs on this integrated dataset followed by graph-based clustering, and projecting of the cells in a 2D space using UMAP was performed. For experiments performed in the same batch on the same chip, normalized datasets were simply merged after filtering for common genes across datasets (*76*). Top 3000 most variable features across merged datasets were identified using *SelectIntegrationFeatures* and were used to calculate the most significant PCs followed by the steps mentioned in the integration workflow.

#### Differential gene expression analysis

Markers of individual clusters (or groups) were defined as the differentially expressed genes between cells belonging to each group versus every other cell using the Wilcoxon rank-sum test as implemented in the function *FindAllMarkers*. Genes with a log fold change >0.25 and a Benjamini-Hochberg–adjusted *P* value <0.05 were considered to be differentially expressed. Pairwise comparison between two clusters of interest was performed using the *FindMarkers* function with similar cutoffs as mentioned.

#### Gene set curation and GSEA

Gene set enrichment scores across individual cells in clusters were quantified using the *AddModuleScore* function in Seurat (*76*). Briefly, a module score for each cell is computed by calculating the average expression of the specified gene set and subtracting the aggregated expression of control genes matched for average expression levels across the dataset. Most gene sets were curated from the literature (*9*, *22*, *53*), while gene lists corresponding to pairwise comparison between T cell compartments were taken from the DESeq2 comparison of our bulk transcriptomic data (data files S1 and S2). The differential expression was ordered according to the |log_2_ fold change| values, and top 200 genes were considered for the gene sets. For in vitro–differentiated cells, cytotoxicity module scores were used to assign cells as low cytotoxicity (≤0), moderate cytotoxicity (>0 ≤ 0.15), and high cytotoxicity (>0.15) (Fig. 6 and fig. S6).

#### Pseudotime trajectory analysis

Integrated single-cell transcriptomic data from ex vivo CD4^+^ T cells and in vitro–differentiated and T_H_1 polarized naïve cells were subjected to a pseudotime trajectory analysis using Monocle3 (*54*). The Seurat-analyzed integrated dataset was converted into *CellDataSet* objects to use them as an input in the Monocle3 framework, and the UMAP generated as part of the Seurat processing pipeline was fed into this *CellDataSet* object. Principal graphs were built without partitions using the *learn_graph* function. Naïve-like cells from the ex vivo CD4 dataset, annotated on the basis of adt_CD45RA, adt_CCR7, and the long-term memory–associated T_CM_ signature score expression pattern (data file S2), were set as the root of the trajectory. Each cell was assigned a pseudotime score, and the average score across clusters was used to order the clusters on the basis of the pseudotime trajectory. Furthermore, the score was used to visualize gene expression pattern changes across the trajectory using the *plot_genes_in_pseudotime* function from the Monocle3 pipeline (*54*).

#### scTCR (V(D)J) repertoire/clonotype analysis

scTCR-seq data were annotated using 10x Genomics’ Ensembl GRCh38 VDJ reference provided as a part of the cellranger multipipeline and further aggregated across datasets using the cellranger vdj aggr pipeline. Clonotypes in each sample were defined using the default clonotype-calling algorithm from the cellranger pipeline (data file S5). Shared clonotypes were defined as clonotypes coming from different cells (≥2) for the same donor. UpSet plots were used for visualizing sharing of clonotypes across clusters or cell types (*79*). Circos plots, as part of the circlize package, were used for visualization of shared clonotypes maintaining the granularity of frequency, cluster, and original dataset information (*80*). The top half of the Circos plot corresponds to the origin of the cells, with the outermost circle representing the dataset origin, while the circle beneath represents the originating cluster. The bottom half of the Circos plot represents the clonotypes being shared with each color representing a unique clonotype. The links between the corresponding cells with their clonotypes are colored on the basis of the clonotype with the arrowhead pointing toward the clonotype and the border of the link colored on the basis of the origin of the cell.

### scATAC-seq

Single-cell open chromatin analysis was performed using the 10x Genomics ATAC version 1.1 kit. Briefly, ex vivo–sorted cells or in vitro–cultured cells were washed thoroughly using 1× PBS, and 55,000 viable cells were pelleted before lysing the cell membrane to isolate nuclei using the lysis buffer (10 mM tris-HCl, pH 7.4, 10 mM NaCl, 3 mM MgCl_2_, 0.1% NP-40, 0.1% Tween 20, 0.01% digitonin, 1% bovine serum albumin, and 1 mM dithiothreitol). Lysis reaction was carried out for an exact 5 min before wash buffer (10 mM tris-HCl, pH 7.4, 10 mM NaCl, 3 mM MgCl_2_, 0.1% Tween 20, 1% bovine serum albumin, and 1 mM dithiothreitol) was added and nuclei were pelleted. Bulk tagmentation reaction was performed using the supplied ATAC enzyme on 25,000 intact nuclei and loaded onto each lane of Chromium NextGEM Chip H. Manufacturers’ recommendations were followed to generate libraries of captured nuclei for open chromatin. Libraries were sequenced on NovaSeq 6000 or NextSeq 2000 (Illumina) as per the kit manufacturer’s recommendation.

### scATAC-seq analysis

Demultiplexed raw ATAC library FASTQ files from each lane of 10x Genomics run were mapped to the human reference genome GRCh38 using 10x Genomics’ cellranger-atac (version 2.1.0) count pipeline. The cell barcode–associated peak matrix (filtered for >20 <10,000 bases) and corresponding fragment files were imported from cellranger run, and all downstream analyses were performed using Signac version 1.13.9 (*35*), unless mentioned otherwise. Barcodes were annotated as cells if at least 500 read pairs passed read filters defined by cellranger version 2.1.0. Further quality control parameters were determined on the basis of the distribution of peak region fragment count, nucleosomal signal, and TSS enrichment for each dataset, and low-quality cells were filtered out: in vitro–differentiated cells—nCount_ATAC <1000 >30,000; ex vivo T_SCM_ cells—nCount_ATAC <1000 >150,000; nucleosomal signal >3 and TSS enrichment <2.5 (*81*).

#### Normalization and integration

Individual peak-barcode matrices were then binarized and normalized using the implementation of the term-frequency inverse-document-frequency transformation (*35*, *82*). Subsequently, singular value decomposition was run (*RunSVD*) on the upper quartile of accessible peaks using the most variable features selected after filtering peaks with counts >10 across all cells [*FindTopFeatures(min.cutoff = 10)*]. Following preprocessing of individual datasets, integration was performed after projecting them into a shared low-dimensional space using reciprocal latent semantic indexing, followed by integration of the individual dataset’s embeddings.

#### Peak calling and cluster generation

Peaks were called on this integrated dataset using MACS2 and filtered for standard chromosomal peaks [*keepStandardChromosomes(pruning.mode = “coarse”)*] and not aligning to ENCODE-defined blacklisted regions. Fresh normalization, as mentioned above, was performed on this integrated dataset for MACS2-called and filtered peaks. Cell clusters were identified using the graph-based clustering method on the SNN graph using 2nd through the 30th dimensions and the integrated latent semantic indexing reduction. UMAP-based visualization was generated using the same components with a Euclidean metric. Resolution was decided empirically: for in vitro–differentiated cells—0.7; for ex vivo T_SCM_ cells—0.4.

#### Gene activity scores

Gene body coordinates for the human genome were imported as an annotation database from TxDb.Hsapiens.UCSC.hg38.knownGene version 3.16.0 from UCSC. Gene activity was then calculated using the function from Signac (GeneActivity) with regions extended 2000 bp upstream of the start site (TSS) to include promoters (*35*). These gene activity scores were then log-normalized and multiplied by the median read counts per cell (nCount_reads).

#### Prediction based on scRNA-seq analysis

scRNA-seq experiments on the same group of cells were performed separately, and cell type annotation (based on clusters for ex vivo T_SCM_, Fig. 3) was transferred onto the scATAC-processed data (query), on the basis of transfer anchors from the most variable features from the scRNA dataset (reference) using canonical correlation analysis reduction (*82*).

#### Gene signature score calculation

Gene sets curated from the literature and our bulk transcriptomic analysis (data file S2) were examined for their accessibility in scATAC datasets. Accessibility scores for each gene set (module) were calculated per cell using the *AddChromatinModule* function in Signac on the basis of peaks linked to the genes in the set (*35*). Briefly, before calculation of signature scores, individual peaks are linked to the activity score for each gene in the dataset (*LinkPeaks*) on the basis of Pearson’s correlation coefficient, and these links are filtered for *P* value <0.05 and *z*-score >0.05 compared to a set of background peaks. Then, the peaks associated with the genes in the given gene set (module) are filtered (*GetLinkedPeaks*) (data file S2; ex vivo T_SCM_ data: CD4-CTL–enriched (cytotoxicity signature)—4007 peaks; CD4-T_EMRA_–enriched (T_EMRA_ signature)—900 peaks; CD4-T_EMRA_ versus T_N_ (200 genes)—1207 peaks; in vitro differentiation data: CD4-CTL–enriched (cytotoxicity signature)—3369 peaks), and deviation for the entire module is calculated per cell using chromVAR on the basis of the human genome BSgenome.Hsapiens.UCSC.hg38 (*83*).

#### Identification of cis-coaccessible networks

The scATAC dataset was leveraged to identify putative cis-regulatory interactions across the gene body, including promoters and enhancers, to understand the regulation of gene expression across clusters. Cicero was used to predict such interactions by examining the coaccessibility of peaks and was run using default parameters (*run_cicero*) (*39*). Cis-coaccessibility networks were then identified on coaccessible peaks using iterative cutoffs defined by the function itself (in vitro–differentiated cells—0.14; ex vivo T_SCM_—0.21).

#### TF motif activity analysis

TF motif activities for each cell were computed using chromVAR on the basis of position weight matrices from the cisbp database (*83*). The motif enrichment analysis was performed in ArchR version 1.0.2 (*84*). The clustering information and the reduced UMAP coordinates for each quality control–passed cell were imported from Signac into ArchR for creating the arrow object, and the same parameters were followed for processing the dataset, as mentioned above. A background peak set controlling for total accessibility and GC content was generated (*addBgdPeaks*), followed by addition of chromVAR motif deviations (*addDeviationsMatrix*) using the cisbp motif set to calculate the enrichment of chromatin accessibility at different TF motif sequences in single cells. To visualize motif deviations, scores were imputed using MAGIC (*85*).

#### ChIP-seq data integration from publicly available datasets

ChIP-seq data for effector TFs were sourced from the literature. The ChIP-seq data for T-bet was from human naïve T_H_1 polarized CD4 cells (GEO: GSE62482 “Replicate 2”) (*41*), EOMES was from human embryonic stem cells differentiated to endodermal fate [Gene Expression Omnibus (GEO): GSE26097 “BoundRegions”] (*42*), and FOSL2 was from human naive CD4^+^ T cells polarized to T_H_17 fate (GEO: GSE174810 “Replicate 1”) (*40*). BED files or narrowPeak files were converted to index-sorted bedGraph file (-cut), which was further converted to BigWig files (*bedgraphtobigwig* from UCSC). Any peaks not already mapped to hg38 were lifted over using the UCSC *LiftOver* tool. Histone modification ChIP-seq was sourced from ENCODE (www.encodeproject.org/). H3K27ac data were downloaded as a signal *P* value BigWig file from the human CD4^+^, alpha-beta memory (CD45RO^+^) T primary cell (ENCFF884NBE from DOI: 10.17989/ENCSR724GUS), while H3K4me1 data were downloaded as a signal *P* value BigWig file from the human CD4^+^, alpha-beta memory (CD45RO^+^) T primary cell (ENCFF334TZP from DOI: 10.17989/ENCSR269SSG) (*38*). The ChIP-seq data were visualized in CoveragePlot for the genomic loci of interest with a default scale.

#### Identification of motifs across genomic loci

The position frequency matrix was imported from the JASPAR2022 vertebrate class database as part of the cellranger-arc-GRCh38-2020-A-2.0.0 reference for TFs of interest (*86*). A motif peak matrix was created on the basis of the position frequency matrix for each TF for the peaks in the dataset of ex vivo T_SCM_ (clusters scATAC-T_SCM_-C6 and scATAC-T_SCM_-C7) and in vitro–differentiated cells (low-, moderate-, and high-cytotoxicity cells) using Signac [*matchMotifs(out = “positions”, p.cutoff = 0.0005)*], with the reference genome BSgenome.Hsapiens.UCSC.hg38 (*35*). Consensus motif–containing peaks with a score cutoff >10 were then visualized using CoveragePlot for genomic regions of interest.

### Quantification and statistical analysis

All statistical tests on flow cytometry data were performed on percentage populations exported from FlowJo using GraphPad Prism version 10.2.2. Student’s paired *t* test was performed to compare between two groups originating from the same donors. All statistical details and population sizes are indicated in the figure legends and Materials and Methods. Population size is described in the figure legends. Data for all flow cytometry analyses or otherwise represented as graphs are provided in data files.
