# Supplementary material for: A distinct subset of stem-cell memory is poised for the cytotoxicity program in CD4+ T cells in humans
Source: Sci Adv. 2026 Jan 7;12(2):eady6423. doi: 10.1126/sciadv.ady6423 (PMC12778050; doi:10.1126/sciadv.ady6423)
Supplement: Supplementary file 1 — Figs. S1 to S6 Table S1 Legends for data files S1 to S6 [file sciadv.ady6423_sm.pdf]

Science Advances
AAAS

Supplementary Materials for

A distinct subset of stem-cell memory is poised for the cytotoxicity program in $CD4^+$ T cells in humans

Raunak Kar *et al.*

Corresponding author: Veena S. Patil, veena@nii.ac.in

Sci. Adv. **12**, eady6423 (2026)
DOI: 10.1126/sciadv.ady6423

The PDF file includes:

Figs. S1 to S6
Table S1
Legends for data files S1 to S6

Other Supplementary Material for this manuscript includes the following:

Data files S1 to S6

Supplementary fig. S1. Transcriptomic and TCR analysis of CD4-T cell memory compartments and CD8-T_{EMRA} subset.

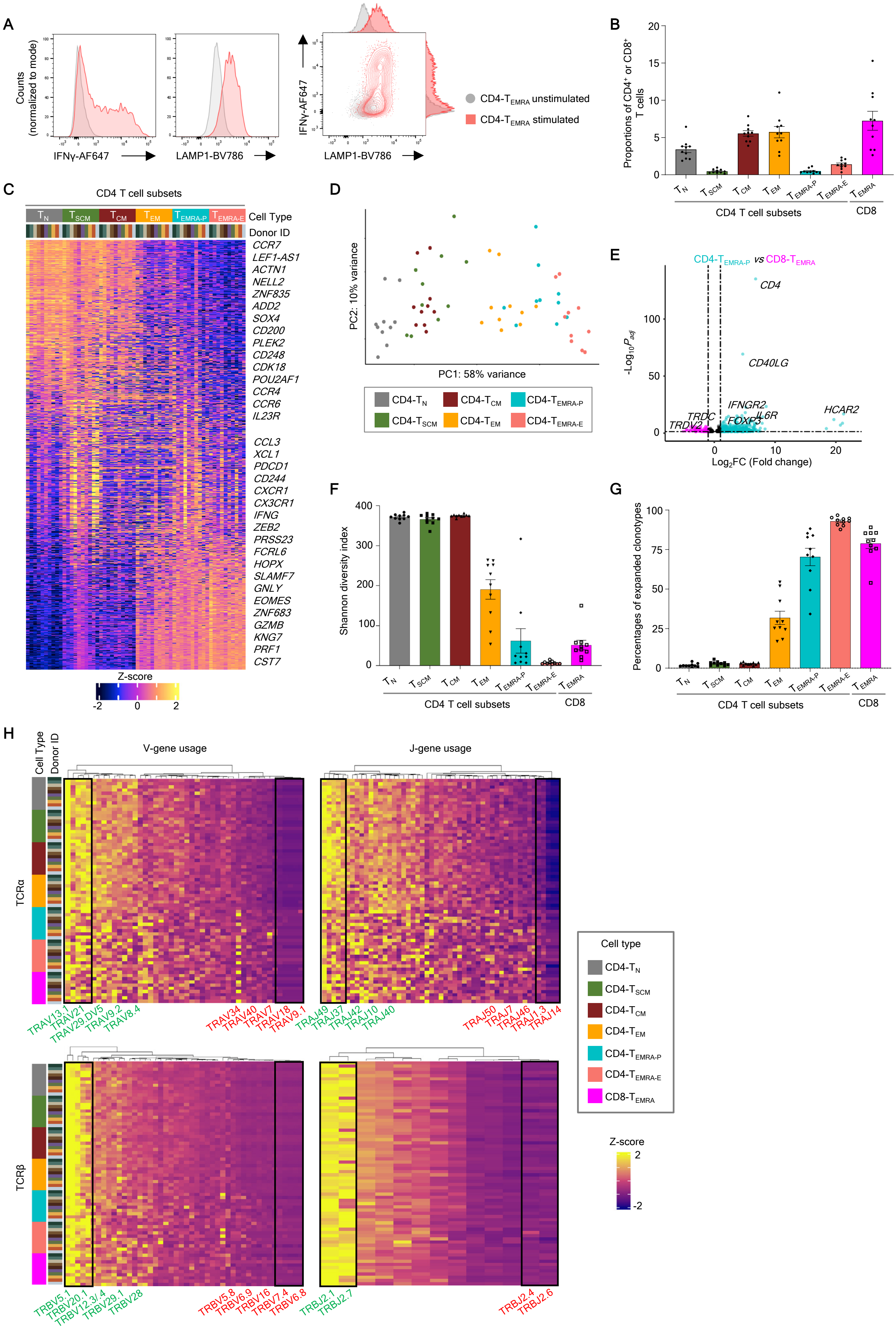

fig. S1. Transcriptomic and TCR analysis of CD4-T cell memory compartments and CD8-T_{EMRA} subset. (A) Representative flow cytometry analysis histogram plots (left) or contour plot (right) for indicated protein expression in CD4-T_{EMRA} compared between unstimulated (grey) and 6 hrs CytoStim stimulated (peach) represented using MFI for single-positives (left) or contour (right) as a function of IFNγ expression (y-axis) and LAMP1 expression (x-axis). (B) Bar graph shows the proportions of indicated memory or naïve T cell subsets within the $CD4^+$ T cell or CD8-T_{EMRA} compartments in the PBMCs of 10 healthy human donors used in the bulk multiomics assays. (C) Heatmap of bulk transcriptomic analysis shows the row-wise z-score of normalized counts of 1000 most variable genes across the indicated T cell memory compartments (indicated at the top) from 10 donors. (D) Principal component analysis (PCA) plot for 1000 most variable genes across the CD4-T cell compartments. (E) Volcano plot for differentially expressed transcripts between CD4-$T_{EMRA\text{-}P}$ and CD8-T_{EMRA} as a factor of -Log_{10}(BH-*adjusted P*) value (y-axis) and Log_2FC(fold change) (x-axis). Significantly differentially expressed transcripts based on DESeq2 (Benjamini-Hochberg P_{adj} <0.05 and log_2FC >1) are colored based on cell type where they are upregulated, turquoise – CD4-$T_{EMRA\text{-}P}$ and pink – CD8-T_{EMRA} (data file S1). (F-G) Bar graphs show the mean Shannon-Weiner diversity index (F) and proportions of expanded clonotypes (≥3) (G) for TCRα clonotypes in the indicated T cell compartment across 10 donors. (H) Heatmap of hierarchically clustered row-wise z-score normalized proportion of V-gene (left) and J-gene (right) usage by the recovered TCRα (top) and TCRβ (bottom). Top and bottom 5 (or 2) V- and J-genes are labelled and highlighted.

Supplementary fig. S2. Multi-omics analysis of $CD4^{+}$ T cell memory subsets.

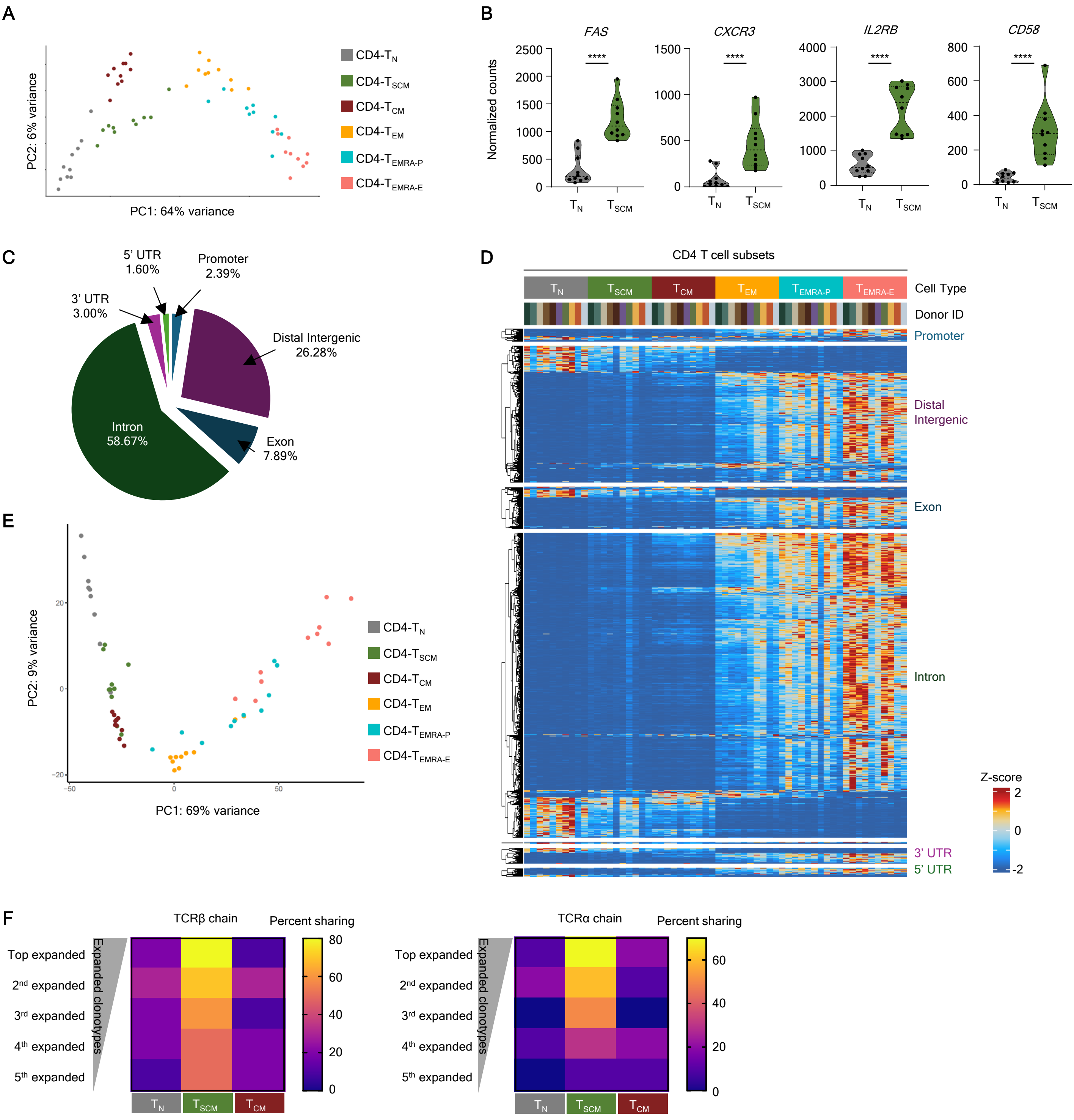

fig. S2. Multi-omics analysis of $CD4^+$ T cell memory subsets. (A) PCA plot for differentially expressed transcripts between T_N and T_{EMRA-E}, (Benjamini-Hochberg P_{adj} value <0.05 and Log_2FC ≥2, 2,772 genes) visualized for all CD4 T cell memory compartments. (B) Violin plots show the normalized counts for indicated transcripts from RNA-Seq analysis compared between T_{SCM} and T_N. Benjamini-Hochberg *P_{adj} <0.05, **P_{adj} <0.01, ***P_{adj} <0.005, ****P_{adj} <0.001 from DESeq2 was used for significance calculation and visualization (data file S1) Each dot represents a donor (n=10). (C) Pie chart shows the contribution of various elements of the gene body to the 2000 most variable peaks across the CD4-T cell memory compartments including naïve. (D) Heatmap of bulk open chromatin analysis shows the row-wise z-score of normalized accessibility counts of hierarchically clustered 2000 most variable peaks across the indicated CD4-T cell memory compartments (indicated at the top) from 10 donors split into the constituent gene body element. (E) Principal component analysis (PCA) plot for 2000 most variable peaks across the CD4-T cell compartments. (F) Heatmap of the proportion of donors (out of 10 donors) sharing the top 5 expanded TCRβ (left) and TCRα (right) clonotypes of T_{EMRA-E} with T_N, T_{SCM}, or T_{CM} subsets.

Supplementary fig. S3. Identification of $T_{SCM\text{-}CTLs}$.

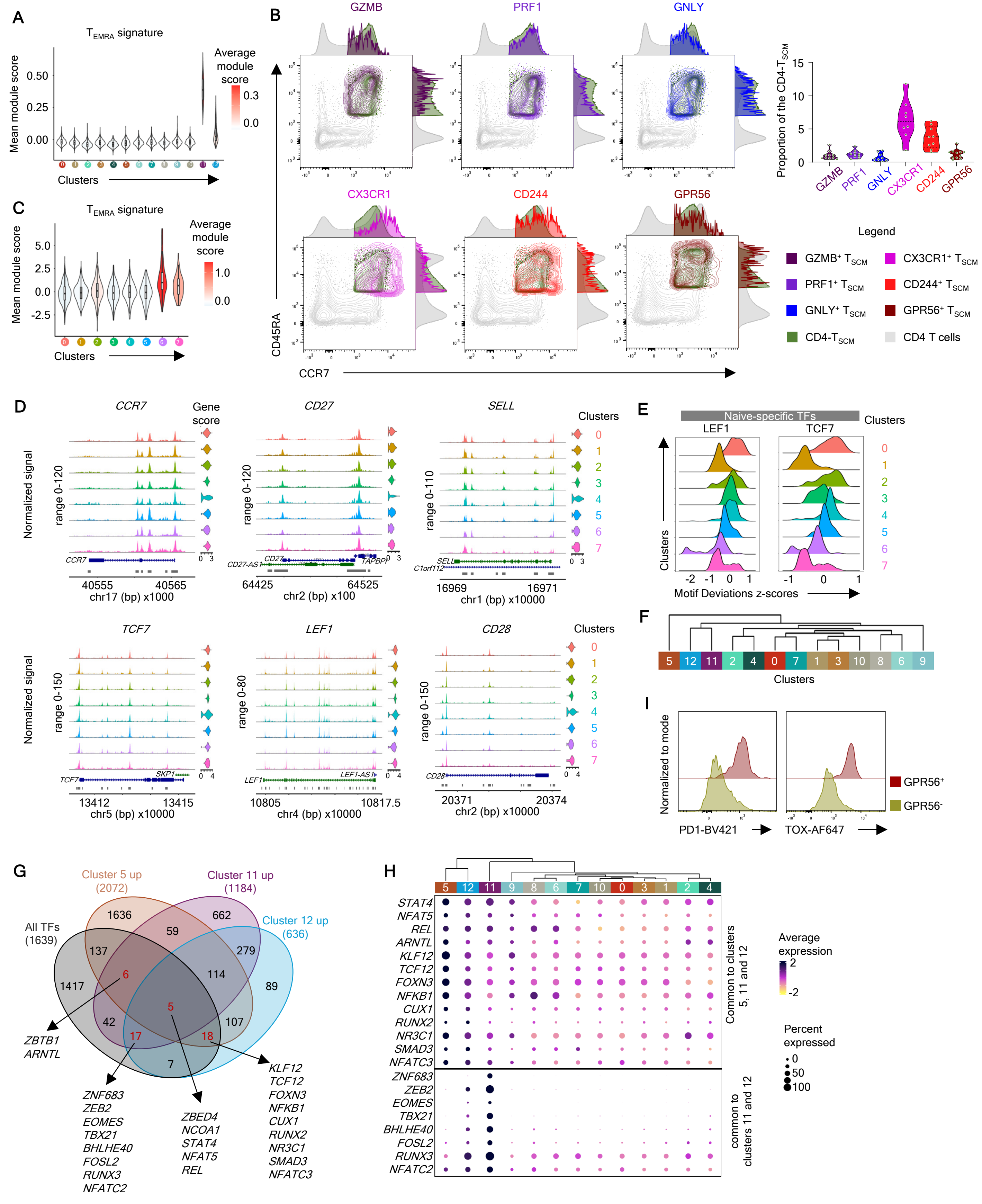

fig. S3. Identification of $T_{SCM-CTLs}$. (A) Violin plot for the mean signature scores for indicated gene module per cluster (x-axis) of scRNA data painted by the average score (data file S2). (B) Flow cytometry contour plots for concatenated PBMC data for 10 to 11 donors showing CCR7 (x-axis) vs CD45RA (y-axis) expression for whole CD4 T cells (light grey), overlaid by entire T_{SCM} compartment (green) and individually $T_{SCM-CTLs}$ based on GZMB, PRF1, GNLY, CX3CR1, CD244 and GPR56 (color mentioned in key) expressions. Bar graph (right) shows the percentage of T_{SCM} cells expressing indicated cytolytic marker protein in 10-11 donors. Each dot represents a donor. (C) Violin plot for the mean accessibility scores (y-axis) for indicated gene modules per cluster (x-axis) of scATAC data painted by the average score; CD4-T_{EMRA} enriched (T_{EMRA} signature) – 900 peaks (data file S2). (D) Chromatin accessibility of the indicated gene-containing genomic loci across clusters, visualized using CoveragePlot with group gene score as violin plots (right). (E) Ridge plot of chromVAR deviation (z-) scores for indicated transcription factors (TFs) across clusters. (F) Hierarchical clustering based on the expression of 1,163 TFs from the human TF database across clusters in the scRNA-seq T_{SCM} data. (G) Venn diagram shows the sharing of DE genes of clusters 5, 11 and 12 (vs rest of the clusters) with known human TFs. The DE gene list obtained from differential expression analysis, based Wilcoxon Rank Sum test with Benjamini-Hochberg P_{adj} value <0.05 and $Log_2FC \geq 0.25$. Examples of overlapping genes are listed. (H) Dot plot of selected differentially expressed TFs across clusters (based on Wilcoxon Rank Sum test with Benjamini-Hochberg P_{adj} value <0.05 and $Log_2FC \geq 0.25$) (data file S4). The clusters are hierarchically clustered based on the expression of shown transcripts. Color represents mean normalized and scaled expression while size represents the percentage of given transcript-expressing cells per cluster. (I) Representative flow cytometry analysis histogram plots for indicated protein expression in $T_{SCM-CTL}$ and non-$T_{SCM-CTL}$ compartments marked by GPR56, represented using mean fluorescence intensity (MFI). For (A, F to H), clusters with <1% of the total cells are not shown (clusters 13 and 14 = 167 and 86 cells respectively).

Supplementary fig. S4. scRNA-Seq and scTCR-Seq analysis of T_{SCM} and T_{EMRA}.

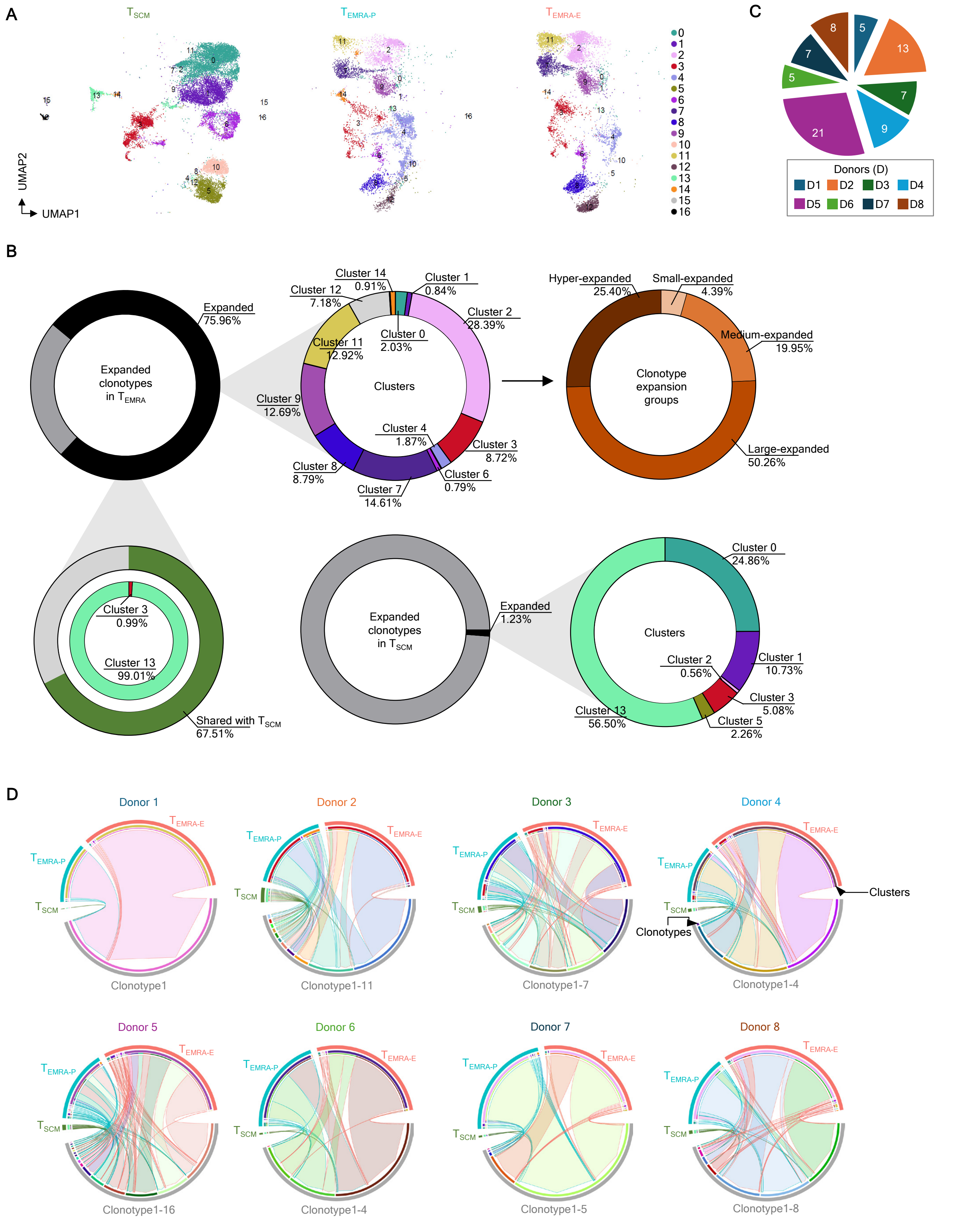

fig. S4. scRNA-Seq and scTCR-Seq analysis of T_{SCM} and T_{EMRA}. (A) 2-D UMAP embedding of 33,041 cells split across T_{SCM}, T_{EMRA-P} and T_{EMRA-E}. (B) Donut plots at the top-left and bottom-middle represent the clonotype expansion status of the T_{EMRA} and T_{SCM} cells respectively. The expanded clonotypes (marked in black) are further split across clusters (top-middle and bottom-right) or shows the sharing of expanded T_{EMRA} clonotypes with T_{SCM} where the inner donut plot showing the distribution across clusters (bottom-left). The donut plot at top-right shows the clonotype expansion status (small expanded ≥3 ≤10, medium expanded >10 ≤100, large expanded >100 ≤500 and hyper expanded >500 ≤1000) of the expanded T_{EMRA} clonotypes. (C) Pie chart shows distribution of 75 shared clonotypes between T_{SCM} and T_{EMRA} (from Fig. 4E) across eight donors (D1-D8). (D) Circos plots show distribution across clusters and categories (T_{SCM}, T_{EMRA-P} and T_{EMRA-E}) of 56 shared expanded clonotypes between T_{SCM} and T_{EMRA} cells split for each donor (D1-D8). The top half of the circos plot corresponds to the origin of the cells, with the outermost circle representing dataset origin while the circle beneath represents the originating cluster. The bottom half of the circos plot represents the clonotypes being shared with each color representing a unique clonotype. The links between the corresponding cells with their clonotypes are colored based on the clonotype with the arrowhead pointing towards the clonotype and the border of the link colored based on the origin of the cell.

Supplementary fig. S5. *in vitro* differentiation and polarization of naïve CD4-T (T_N) cells.

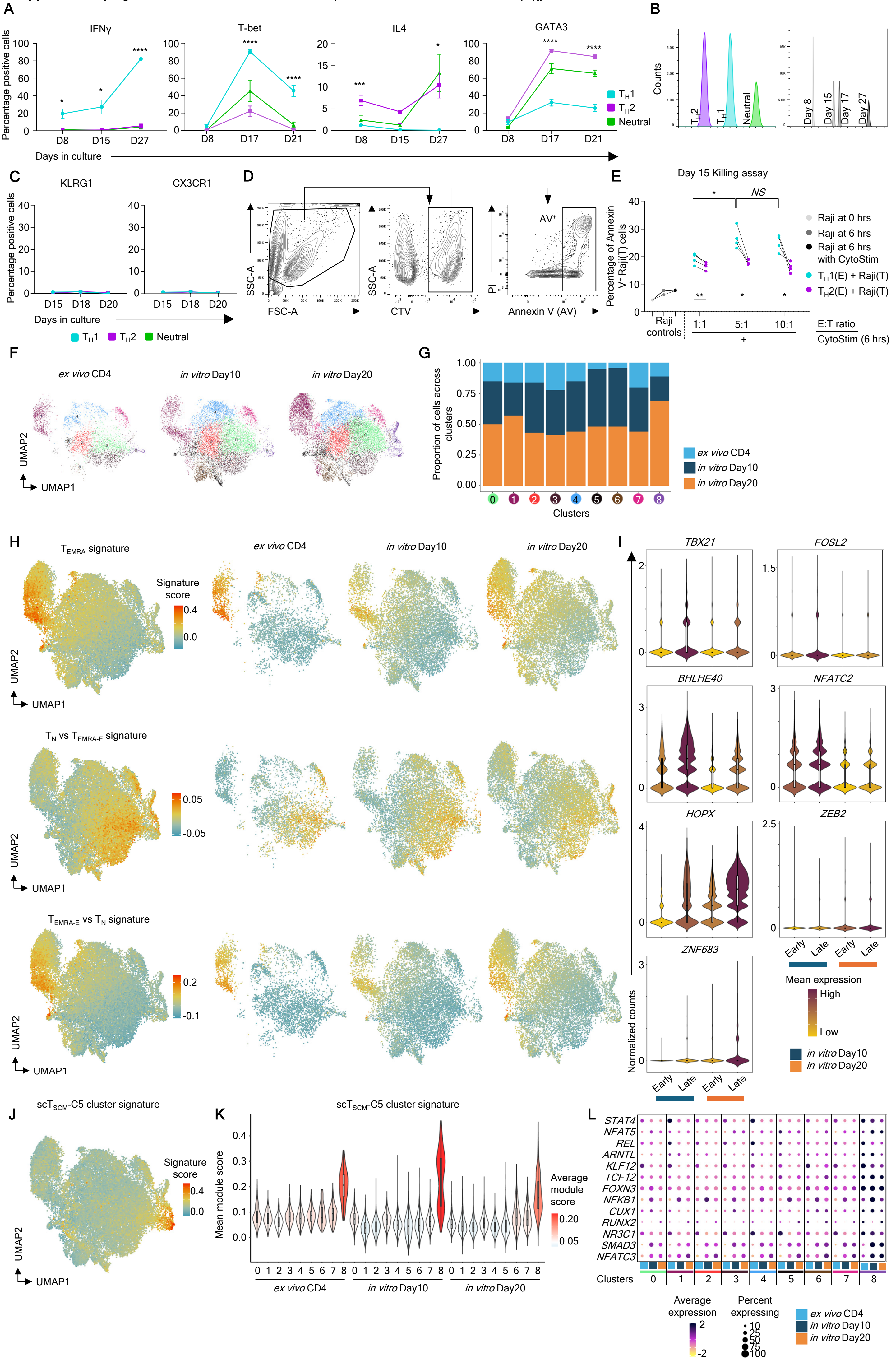

fig. S5. *in vitro* differentiation and polarization of naïve CD4-T (T_N) cells. (A) Summary flow cytometry data of indicated cytokines (IFNγ and IL4) and transcription factors (T-bet and GATA3) across days comparing T_H1-(n=6), T_H2-(n=6) or neutral-(n=3) polarized cells. Error bars for each point represent mean ± SEM. Y-axis represents percentage of live-singlet gated cells expressing the mentioned protein while x-axis represents days in culture (left). *P <0.05, **P <0.01, ***P <0.005, ****P <0.001 from Student's paired two-tailed T test comparing T_H1 vs T_H2 polarizing conditions. (B) Concatenated flow cytometry data for 132,582 live-singlet gated *in vitro* differentiated and T_H1-, T_H2- and neutral- polarized cells (n=6, 6 and 3 donors respectively) from count normalized samples across donors from days 8, 15, 17 and 27 of culture, split based on polarization conditions and days, represented by count histogram. (C) Summary flow cytometry data of indicated cell surface proteins across days comparing T_H1-(n=6) or T_H2-(n=6) polarized cells. Error bars for each point represent mean ± SEM. Y-axis represents percentage of live-singlet gated cells expressing the mentioned protein while x-axis represents days in culture (left). (D) Representative flow cytometry contour plots show the gating strategy used to calculate Annexin V^+ cells in the killing assay. (E) Matched scatter plot shows the percentage of Annexin V^+ cells in the indicated groups at day 15 killing assay. T:target cells (Raji); E:effector cells (T_H1- or T_H2- polarized and differentiated cells at day 15, n=4). *P NS*>0.05, *<0.05, **<0.01, ***<0.005, ****<0.001 from Student's paired two-tailed T test. (F) Integrated 2-D UMAP embedding of 26,815 QCed-cells from *ex vivo* CD4 and *in vitro* differentiated and T_H1 polarized T cells, colored by clusters split by origin. (G) Stacked bar graph of proportion of cells (y-axis) from each dataset across different clusters (x-axis). (H) 2-D UMAP projection painted by indicated gene signatures either together (left) or split by original dataset (right) (data file S2). (I) Violin plots show the mean expression across groups of early (clusters 3, 0, 7 and 8) and late (clusters 2, 6, 4, 5 and 1) clusters split between D10 and D20 for indicated transcripts. (J-K) Enrichment analysis of cluster 5 (of scRNA T_{SCM})-specific gene sets visualized either using 2-D UMAP projection (J) or violin plots showing module score split based on origin and clusters (K) (data

file S4). (L) Dot plot for the expression of selected TFs across clusters split by origin. Color represents mean normalized and scaled expression while size represents the percentage of given transcript-expressing cells per cluster.

Supplementary fig. S6. T_H1 polarized cells undergo gradual change to acquire cytotoxicity program.

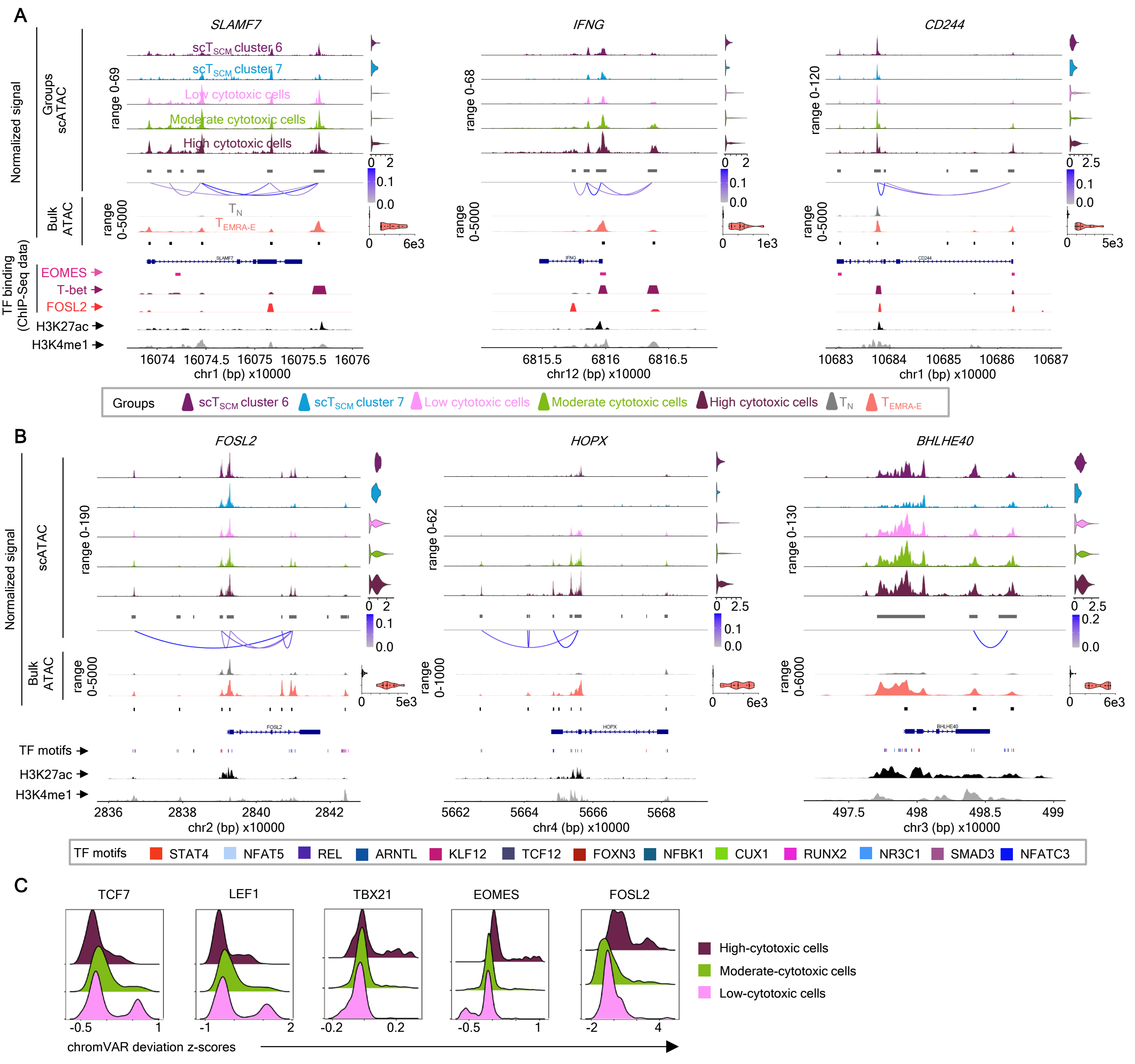

fig. S6. T_H1 polarized cells undergo gradual change to acquire cytotoxicity program. (A-B) Single-cell (T_{SCM} and differentiated cells) or bulk (CD4-T_N and T_{EMRA-E}, n=10, aggregated) ATAC-Seq analysis show the chromatin accessibility of the indicated gene-containing genomic loci, visualized using CoveragePlot with cluster-wise group gene score for single-cell open chromatin data or normalized gene expression (transcriptomic data) for bulk (T_N and T_{EMRA-E}) data as violin plots (right) and significant co-accessible peaks connected via links colored based on scores (graded blue scale, cut-off <0.05). ENCODE sourced histone modification marks of H3K27ac and H3K4me1 are shown. Publicly available ChIP-Seq data for T-bet, EOMES and FOSL2 for the corresponding genomic loci shown as colored peak plots (A). TF of interest (different colors) having consensus motifs in peaks from scATAC data of *in vitro* differentiated cells represented as peak plot (B). (C) Ridge plot of chromVAR deviation (z-) scores for indicated TFs in *in vitro* differentiated cells across categories (low-, moderate-, high-).

SUPPLEMENTARY TABLES

Table S1. List of reagents used in study.

**Fluorochrome antibodies / reagents used in study**				
**Antigen**	**Fluorochrome**	**Clone**	**Catalog number**	**Company**
CD3	AF700	UCHT1	300424	BioLegend
CD3	APC Cy7	UCHT1	300426	BioLegend
CD3	BUV395	SK7	564001	BD Biosciences
CD3	PE Cy7	UCHT1	300420	BioLegend
CD4	FITC	OKT-4	317408	Bio Legend
CD4	APC Cy7	RPA-T4	300518	BioLegend
CD4	PE Cy7	SK3 (Leu3a)	557852	BD Biosciences
CD4	PE Dazzle	A161A1	357412	BioLegend
CD8A	FITC	RPA-T8	301006	BioLegend
CD8A	PE Dazzle	RPA-T8	301058	BioLegend
CD45RA	BV785	Hi100	304140	BioLegend
CD45RA	APC	HI100	304112	BioLegend
CD45RA	BV421	5H9	569620	BD Biosciences
CD45RA	BV650	HI100	304136	BioLegend
CD45RA	BV711	HI100	304138	BioLegend
CCR7	APC	G043H7	353214	BioLegend
CCR7	PE	G043H7	353204	BioLegend
CCR7	PE Cy7	G043H7	353226	BioLegend
CD95	BV421	DX2	305624	BioLegend
CD95	BV605	DX2	305628	BioLegend
CD127	BV650	A019D5	351326	BioLegend
CD127	APC	A019D5	351316	BioLegend
CD107a(LAMP-1)	BV785	H4A3	328644	BioLegend
CD244 (2B4)	PE Dazzle	C1.7	329522	BioLegend
CX3CR1	FITC	2A9-1	341606	BioLegend

CX3CR1	BV650	2A9-1	341626	BioLegend
EOMES	PE Cy5.5	WD1928	35-4877-42	eBiosciences
EOMES	PE Cy7	WD1928	25-4877-42	eBiosciences
GPR56	PE	CG4	358204	BioLegend
GPR56	PE Cy7	CG4	358206	BioLegend
Granulysin	APC	DH2	348010	BioLegend
Granzyme B	APC	QA16A02	372204	BioLegend
Granzyme B	FITC	QA16A03	372206	BioLegend
IFNg	AF647	4S.B3	502516	BioLegend
IFNg	FITC	4S.B3	502506	BioLegend
IL-4	PE	8D4-8	12-7049-42	ebiosciences
KLRG1	PE	13A2	12-9487-42	ebiosciences
Perforin	BV421	B-D48	353307	BioLegend
Perforin	PE	B-D48	353304	BioLegend
GATA3	AF647	16E10A23	653810	BioLegend
T-bet	BV421	4B10	644816	BioLegend
TCF1 (TCF7)	PE	7F11A10	655208	BioLegend
PD1	BV421	EH122H7	329920	BioLegend
TOX	AF647	NAN448B	568356	BD Biosciences
4',6-diamidino-2-phenylindole (DAPI)			D1306	Invitrogen
Propidium Iodide (PI)			P1304MP	Invitrogen
Zombie Aqua Fixable Viability Kit	BV510		423102	BioLegend
CellTrace Violet Cell Proliferation Kit	BV421		C34557	Invitrogen
Annexin V AF488 Conjugate for Apoptosis Detection	AF488		A13201	Invitrogen

CITE-Seq antibodies used in study					
**Antigen**	**Product name**	**Clone**	**Identifier Sequence**	**Catalog Number**	**Company**
Fas (CD95)	TotalSeqTM-C 0156 anti-human CD95 (Fas)	DX2	CCAGCTCATTAGAGC	305651	Biolegend
CCR7	TotalSeqTM-C 0148 anti-human CD197 (CCR7)	G043H7	AGTTCAGTCAACCGA	353251	Biolegend
IL7Ra (CD127)	TotalSeqTM-C 0390 anti-human CD127 (IL7Ra)	A019D5	GTGTGTTGTCCTATG	351356	Biolegend
CD45RA	TotalSeqTM-C 0063 anti-human CD45RA Antibody	HI100	TCAATCCTTCCGCTT	304163	Biolegend
CD4	TotalSeqTM-C 0045 anti-human CD4	SK3	GAGGTTAGTGATGGA	344651	BioLegend
CD8	TotalSeqTM-C 0046 anti-human CD8	SK1	GCGCAACTTGATGAT	344753	BioLegend
CD298; β2M	TotalSeqTM-C 0251 anti-human Hashtag 1	LNH-94; 2M2	GTCAACTCTTTAGCG	394661	Biolegend
CD298; β2M	TotalSeqTM-C 0252 anti-human Hashtag 2	LNH-94; 2M2	TGATGGCCTATTGGG	394663	Biolegend
CD298; β2M	TotalSeqTM-C 0253 anti-human Hashtag 3	LNH-94; 2M2	TTCCGCCTCTCTTTG	394665	Biolegend
CD298; β2M	TotalSeqTM-C 0254 anti-human Hashtag 4	LNH-94; 2M2	AGTAAGTTCAGCGTA	394667	Biolegend
CD298; β2M	TotalSeqTM-C 0255 anti-human Hashtag 5	LNH-94; 2M2	AAGTATCGTTTCGCA	394669	Biolegend
CD298; β2M	TotalSeqTM-C 0256 anti-human Hashtag 6	LNH-94; 2M2	GGTTGCCAGATGTCA	394671	Biolegend
CD298; β2M	TotalSeqTM-C 0257 anti-human Hashtag 7	LNH-94; 2M2	TGTCTTTCCTGCCAG	394673	Biolegend
CD298;	TotalSeqTM-C 0258 anti-	LNH-94;	CTCCTCTGCAATTAC	394675	Biolegend

β2M	human Hashtag 8	2M2			
CD298; β2M	TotalSeqTM-C 0259 anti-human Hashtag 9	LNH-94; 2M2	CAGTAGTCACGGTCA	394677	Biolegend
CD298; β2M	TotalSeqTM-C 0260 anti-human Hashtag 10	LNH-94; 2M2	ATTGACCCGCGTTAG	394679	Biolegend
CD298; β2M	TotalSeqTM-C 0262 anti-human Hashtag 12	LNH-94; 2M2	TAACGACCAGCCATA	394683	Biolegend

**Culture reagents used in study**			
**Reagent**	**Clone**	**Catalog Number**	**Company**
αCD3/αCD28 DynaBeads Human T cell activator		11131D	Invitrogen
Gentamicin		15750060	Invitrogen
Recombinant Human IL-2		589102	Biolegend
Recombinant Human IL-4		204-IL-010/CF	R&D Systems
Recombinant Human IL-12		219-IL-005/CF	R&D Systems
Recombinant Human IFN-g		BMS303	eBiosciences
Anti-human IL-4 mAb	3007	MAB304	R&D Systems
Anti-human IFN-g mAb	NIB42	554547	BD Biosciences
Anti-human IL-12 p70 mAb	24910	MAB219	R&D Systems
Anti-human IL-17A mAb	eBio64CAP17	16-7178-85	eBiosciences
CytoStim		130-092-173	Miltenyi Biotec

Other Supplementary Material for this manuscript includes the following:

Supplementary Data files S1 to S6

Supplementary Data file S1 (Microsoft Excel Format): List of differentially expressed genes from bulk RNA-Seq analysis.

Supplementary Data file S2 (Microsoft Excel Format): List of gene sets used for enrichment analysis.

Supplementary Data file S3 (Microsoft Excel Format): Bulk TCRα and TCRβ repertoire analysis of T cell memory subsets.

Supplementary Data file S4 (Microsoft Excel Format): List of differentially expressed genes between clusters in single-cell transcriptomic analysis of T_{SCM} subset.

Supplementary Data file S5 (Microsoft Excel Format): Single-cell TCR repertoire analysis of T cells.

Supplementary Data file S6 (Microsoft Excel Format): List of differentially expressed genes between clusters in single-cell transcriptomic analysis of *in vitro* polarized naïve T cells at Day10 and Day20.

Source Data (Microsoft Excel Format)
